# Cooperative bundling by fascin generates actin structures with architectures that depend on filament length

**DOI:** 10.3389/fcell.2022.974047

**Published:** 2022-09-02

**Authors:** Laura A. Sherer, Naomi Courtemanche

**Affiliations:** Department of Genetics, Cell Biology and Development, University of Minnesota, Minneapolis, MN, United States

**Keywords:** actin, fascin, polymerization, bundling, formin

## Abstract

The assembly of actin-based structures with precisely defined architectures supports essential cellular functions, including motility, intracellular transport, and division. The geometric arrangements of the filaments within actin structures are stabilized via the association of crosslinking proteins, which bind two filaments simultaneously. Because actin polymerization and crosslinking occur concurrently within the dynamic environment of the cell, these processes likely play interdependent roles in shaping the architectures of actin-based structures. To dissect the contribution of polymerization to the construction of higher-order actin structures, we investigated how filament elongation affects the formation of simple, polarized actin bundles by the crosslinking protein fascin. Using populations of actin filaments to represent distinct stages of elongation, we found that the rate of bundle assembly increases with filament length. Fascin assembles short filaments into discrete bundles, whereas bundles of long filaments merge with one another to form interconnected networks. Although filament elongation promotes bundle coalescence, many connections formed between elongating bundles are short-lived and are followed by filament breakage. Our data suggest that initiation of crosslinking early in elongation aligns growing filaments, creating a template for continued bundle assembly as elongation proceeds. This initial alignment promotes the assembly of bundles that are resistant to large changes in curvature that are required for coalescence into interconnected networks. As a result, bundles of short filaments remain straighter and more topologically discrete as elongation proceeds than bundles assembled from long filaments. Thus, uncoordinated filament elongation and crosslinking can alter the architecture of bundled actin networks, highlighting the importance of maintaining precise control over filament length during the assembly of specialized actin structures.

## Introduction

The actin cytoskeleton dynamically assembles into discrete structures to support essential cellular functions, including motility, intracellular transport, and division. Construction of actin-based structures requires the polymerization of actin monomers into filaments, and the incorporation of these filaments into networks with precisely defined architectures (Blanchoin et al., 2014). The specific geometric arrangements of the filaments within actin structures are stabilized via the association of crosslinking proteins, which bind two filaments simultaneously (Michelot and Drubin, 2011; Blanchoin et al., 2014; Lappalainen, 2016; Pollard, 2016). The molecular properties of crosslinking proteins specify filament spacing, orientation, and rotational freedom, and thus confer a unique set of mechanical properties to each structure (Bartles, 2000; Winkelman et al., 2016; Svitkina, 2018).

Crosslinking is a dynamic process that requires the assembly of actin filaments (Blanchoin et al., 2014). In the cellular environment, actin polymerization and crosslinking occur simultaneously (Mallavarapu and Mitchison, 1999; Vavylonis et al., 2006; Tang et al., 2015)), so these processes likely play interdependent roles in shaping the architectures and mechanical properties of actin-based structures. Recent studies have revealed that the rate at which actin filaments are generated influences the architecture of crosslinked networks assembled *in vitro* by alpha-actinin (Falzone et al., 2012, 2013). Alpha-actinin is a flexible crosslinker that assembles actin filaments into bundles that are characterized by a relatively wide inter-filament spacing of 35 nm and exhibit mixed polarity (Hampton et al., 2007). Increasing the rate of actin polymerization shifts the morphology of the structures generated by alpha-actinin from a population of sparse, thick bundles to a dense network of interconnected filaments (Falzone et al., 2012, 2013). However, it remains unknown how the assembly of crosslinked actin structures evolves over the course of polymerization, and whether the length at which filaments initially become crosslinked influences the overall topology of the resultant actin network.

To answer these questions, we investigated how polymerization affects the assembly of simple, polarized actin bundles by the crosslinking protein fascin. Fascin is a monomeric actin-binding protein that localizes to protrusive cellular structures such as filopodia, invadopodia, and dendritic cell membrane extensions (Vignjevic et al., 2006; Li et al., 2010; Yamakita et al., 2011; Yang et al., 2013; Van Audenhove et al., 2016). These thin projections push against and deform the plasma membrane as they assemble and elongate outwards from the cell body (Mallavarapu and Mitchison, 1999), enabling them to play major roles in cellular motility, guidance, and invasion (Mattila and Lappalainen, 2008; Svitkina, 2018). The actin filaments that are assembled into protrusive structures are polymerized by formins and Ena/VASP family proteins (Schirenbeck et al., 2005; Breitsprecher et al., 2008; Lizárraga et al., 2009; Mellor, 2010; Jaiswal et al., 2013; Arthur et al., 2021). These filaments are assembled into ordered arrays by fascin, which bundles actin filaments by binding cooperatively along their lengths (Bryan and Kane, 1978; Yamashiro-Matsumura and Matsumura, 1985; Vignjevic et al., 2006). Fascin’s compact three-dimensional structure specifies a relatively short inter-filament distance of 8 nm (Sedeh et al., 2010; Jansen et al., 2011), and its preference for binding filaments that are aligned in parallel orientations confers polarity to the bundles that it assembles *in vitro* (Courson and Rock, 2010). As a result, filament elongation primarily occurs at one end of each fascin-bound bundle (Jaiswal et al., 2013; Winkelman et al., 2014), consistent with the extension of protrusive structures in a single direction, away from the cell body. This topological feature facilitates direct measurement of bundle elongation within dynamically assembling filament networks.

Because protrusive structures elongate as they are assembled (Mallavarapu and Mitchison, 1999), we focused specifically on the effects of filament elongation on fascin-mediated bundling. Using a formin to generate populations of actin filaments with lengths representing distinct stages of elongation, we found that the rate of bundle assembly increases with filament length. We further observed that fascin assembles short filaments into topologically discrete bundles, whereas bundles of long filaments expand to form interconnected networks by incorporating additional filaments and forming stable, inter-bundle connections. Introducing actin monomers into reactions containing bundles of short filaments promotes their elongation and enables inter-bundle crosslinking. However, most connections formed between elongating bundles are short-lived and are followed by filament breakage at or near the initial site of crosslinking. Taken together, our data reveal that initiation of filament bundling early in elongation (i.e., when filaments are short) establishes a template that constrains the flexibility of the bundle. This increases the resistance of the bundle to changes in curvature that are required to form stable, interconnected networks. As a result, bundles of short filaments remain straighter as they elongate than bundles assembled from long filaments. Thus, uncoordinated filament elongation and crosslinking alters the morphology of actin bundles assembled by fascin, highlighting the importance of maintaining precise regulation of filament length during the assembly of specialized actin structures.

## Materials and methods

### Protein purification

Actin was extracted from chicken skeletal muscle acetone powder and purified by one cycle of polymerization and depolymerization (Spudich and Watt, 1971). Monomers were separated from oligomers and filaments via gel filtration using Sephacryl S-300 resin (GE Healthcare) in G-buffer (2 mM Tris, pH 8.0, 0.2 mM ATP, 0.5 mM DTT, 0.1 mM CaCl_2_). Fluorescent actin for experiments with elongating bundles was generated by labeling Cysteine 374 with Oregon Green 488 iodoacetamide (Thermo Fisher Scientific) (Kuhn and Pollard, 2005). A construct encoding the Formin Homology 1 and 2 (FH1 and FH2) domains of the formin Cdc12p (residues 882–1,375; henceforth referred to as “Cdc12p”) was cloned into the pGEX-4T-3 vector and expressed with an N-terminal glutathione S-transferase (GST) tag and a C-terminal polyhistidine tag in BL21-CodonPlus (DE3)-RP cells (Agilent Technologies). Cdc12p purification was performed as previously described (Sherer et al., 2018).

Human fascin-1 was expressed in BL21 (DE3)pLysS cells from a pET21a plasmid that was modified to encode an N-terminal GST tag and a Tobacco Etch Virus (TEV) protease cleavage recognition sequence. Transformants were grown in 1 L of LB broth, induced at OD_600_ ∼0.6 with 0.5 mM IPTG, and shaken at 16°C overnight. To purify fascin, resuspended cell pellets were sonicated in lysis buffer (50 mM Tris, pH 8.0, 500 mM NaCl, 1 mM DTT) and centrifuged (∼31,000 x *g*, 4°C) for 40 min to isolate soluble cell components. The lysate was incubated with glutathione agarose resin (pH 8.0) for 1 hour with rotation at 4°C and transferred into a glass column. The resin was washed with low-salt buffer (50 mM Tris, pH 8.0, 100 mM NaCl, 1 mM DTT), and the protein was eluted with 100 mM GSH (pH 8.0) in low-salt buffer. The eluted protein was incubated with ∼2–5 µM maltose-binding protein (MBP)-tagged TEV protease at 4°C overnight to remove the GST tag. The protein was dialyzed into low-salt buffer, followed by a 1-h incubation with glutathione agarose resin to remove the cleaved GST tag. After collecting the flowthrough, the TEV protease was removed by applying the sample to an amylose column and collecting the flowthrough. Pure protein samples were concentrated using 30,000 molecular-weight cutoff spin columns (MilliporeSigma), dialyzed into KMEI buffer (50 mM KCl, 1 mM MgCl_2_, 1 mM EGTA, 10 mM imidazole, pH 7.0), flash-frozen, and stored at -80 °C. The extinction coefficient used to calculate fascin concentration was 68,465 M^−1^ cm^−1^.

### Co-sedimentation assays

Ca^2+^-actin monomers were converted to Mg^2+^-actin via the addition of 0.05 mM MgCl_2_ and 0.2 mM EGTA. Samples containing 15 μM Mg^2+^-actin monomers were polymerized in KMEI buffer for 45 min at 22°C in the presence or absence of 250 nM Cdc12p to generate short and long filaments, respectively. Polymerized samples were diluted to 2 µM actin and incubated with a range of fascin concentrations for 1 h at 22°C. Samples were centrifuged for 30 min at 10,000 x *g* (long filaments) or 20,000 x *g* (short filaments). Supernatants and pellets were separated and analyzed via SDS-PAGE. Gel band densities were quantified using ImageJ and normalized by molecular weight. The intensity of the fascin band in the pelleted fraction was divided by the intensity of the actin band in the pelleted (i.e., bundled) fraction for each reaction and plotted as a function of the fascin concentration. Data were fit with the McGhee-von Hippel equation (McGhee and von Hippel, 1974; De La Cruz, 2005; Andrianantoandro and Pollard, 2006) as follows:

$$y=0.5*\left(1+\frac{-1+K_{a}x\omega }{\sqrt{1+\;K_{a}x\left(4+\omega \left(-2+K_{a}x\omega \right)\right)}}\right)$$ (1)

where y is the binding density of fascin on actin bundles, K_a_ is the association constant, x is the concentration of fascin, and ω is the cooperativity parameter. Dissociation (K_d_ = 1/K_a_) and cooperativity constants were measured using the fits from at least three independent experiments.

Co-sedimentation assays with phalloidin- or Cdc12p-bound long actin filaments were performed as described above, except that filaments polymerized in reactions containing 15 μM Mg^2+^-actin monomers were diluted to a concentration of 2 µM actin and incubated with either 2 µM FITC-phalloidin or 100 nM Cdc12p for 20 min at 22°C prior to the introduction of fascin.

### Bundling assays

Samples containing 2 μM Mg^2+^-actin monomers were polymerized in KMEI buffer for 1 h at 22°C in the absence or presence of 85 nM Cdc12p to generate long or short filaments. Following polymerization, actin filaments were incubated with 4 µM FITC-phalloidin for 20 min. Samples were diluted to 10 nM actin (long filaments) or 2 nM actin (short filaments) in microscopy buffer (10 mM imidazole, pH 7.0, 50 mM KCl, 1 mM MgCl_2_, 1 mM EGTA, 50 mM DTT, 0.2 mM ATP, 15 mM glucose, 20 μg/ml catalase, 100 μg/ml glucose oxidase, 0.5% (w/v) methylcellulose (4,000 cP at 2%)). These dilutions yield identical concentrations of filaments for samples containing short and long filaments. Samples were transferred to imaging surfaces that were constructed by placing Scotch tape (3M) around the perimeter of a 4.5 mm × 4.5 mm region of glass coverslips (#1.5, 22 mm × 50 mm) as described previously (Zweifel et al., 2021). After collecting baseline images of the samples, fascin was introduced to initiate bundling. Reactions were visualized at 10–30 s intervals for 20–60 min by through-objective total internal reflection fluorescence (TIRF) microscopy on an Olympus Ti83 motorized microscope equipped with a CellTIRF system using a 60x, 1.49 NA objective and a 488-nm laser. Images were acquired using a Hamamatsu C9100-23B ImagEM X2 EMCCD camera and CellSens Dimension software (Olympus).

For assays with elongating bundles, short filaments were generated by polymerizing 2 μM Mg^2+^-ATP-actin with 85 nM Cdc12p for 1 h at 22°C and labeled with an equimolar concentration of FITC-phalloidin for 20 min. Samples were diluted to a concentration of 0.25 µM actin and incubated with 1 µM fascin for 30 min to generate bundles. Bundles were diluted to concentrations corresponding to 5 nM actin in microscopy buffer containing 10 µM *S. pombe* profilin, 1 µM fascin, and 0.75 or 1.5 μM Mg^2+^-actin monomers (5% Oregon Green-labeled). Reactions were visualized by TIRF microscopy.

### Microscopy image analysis

Fluorescence micrographs were analyzed using ImageJ and custom-built MATLAB programs developed in-house. Actin filaments and bundles were detected from background-subtracted and noise-filtered micrographs using MATLAB’s global or adaptive thresholding algorithms. To quantify filament lengths, actin filaments were skeletonized using MATLAB’s bwskel function and overlapping filaments were identified and resolved with user input prior to length quantification. Filament lengths were quantified for three independent replicates using at least five fields of view per replicate. Single exponential fits were applied to filament length distributions. For each exponential distribution, the fraction of filaments (*f*_*i*_) with length *l* was determined by the following relation:

$$f_{i}=\lambda e^{\left(-\lambda l_{i}\right)}$$ (2)

where the mean length is 1/_λ_ and the variance (*l*_*i*_) is 1*/*_λ_^2^.

Bundles were identified computationally by setting a threshold fluorescence intensity corresponding to the signal produced by two overlapping filaments. Accurate bundle detection was confirmed visually. Bundling was quantified using the following equation:

$$Fraction\;Bundled=\;\frac{F_{Bundled}\;}{F_{Total}}$$ (3)

where F_bundled_ is the sum of the individual pixel fluorescence intensities above the set bundle threshold and F_total_ is the sum of all pixel intensities within skeletonized objects. This metric accounts for the incorporation of additional filaments into preexisting bundles as reactions progress and any variability in the total filamentous actin present across samples. The average background fluorescence was subtracted from all fluorescence measurements to enable accurate comparisons across samples. To account for photobleaching, bundle intensity thresholds were adjusted using changes in the fluorescence intensity of single filaments over time. Time courses of bundling were fit using the following single (2) or double (3) exponential equations:

$$Fraction\;Bundled=\;Ae^{-bt}+c$$ (4)

$$Fraction\;Bundled=\;A_{1}e^{-b_{1}t}+A_{2}e^{-b_{2}t}+c$$ (5)

where A represents the amplitude, b is the bundling rate, and c is the offset. For samples that did not attain equilibrium during the observation period, curve fits were constrained using the average amplitude of reactions with equivalent fascin concentrations. Bundling reactions were performed in triplicate with up to three 80 μm × 80 µm imaging fields analyzed per experiment.

## Results

Actin polymerization and bundling are dynamic reactions that take place simultaneously during the assembly of higher-order actin structures (Mallavarapu and Mitchison, 1999; Vavylonis et al., 2006; Tang et al., 2015). As a result, monitoring the progress of each reaction is complicated by contributions from the other. To investigate the effects of filament elongation on the mechanism of fascin-mediated bundling, we sought to simplify our reactions by systematically controlling the progress of the elongation reaction. To do so, we used a formin to assemble populations of actin filaments with defined lengths. We used these preassembled filaments to represent progressive stages of elongation, with longer filaments representing later time points.

We selected the fission yeast formin Cdc12p as a representative formin for our assays because it decreases the filament elongation rate by 99% in the absence of profilin (Kovar et al., 2003, 2006). The extremely slow elongation of filaments bound by Cdc12p enables us to directly control filament length by modulating the concentrations of actin and formin (Zweifel et al., 2021). Importantly, unlike the mammalian formins FHOD1 and mDia2, which also possess slow elongation activities, and Daam1 and FMNL2, which polymerize actin filaments that are incorporated into protrusive structures (Harris et al., 2006; Esue et al., 2008; Jaiswal et al., 2013; Schönichen et al., 2013), Cdc12p does not bundle or bind along the lengths of actin filaments when present at concentrations exceeding 100 nM (Scott et al., 2011). Cdc12p is therefore ideally suited for our assays, which require relatively high formin concentrations to generate short actin filaments.

**FIGURE 1:**
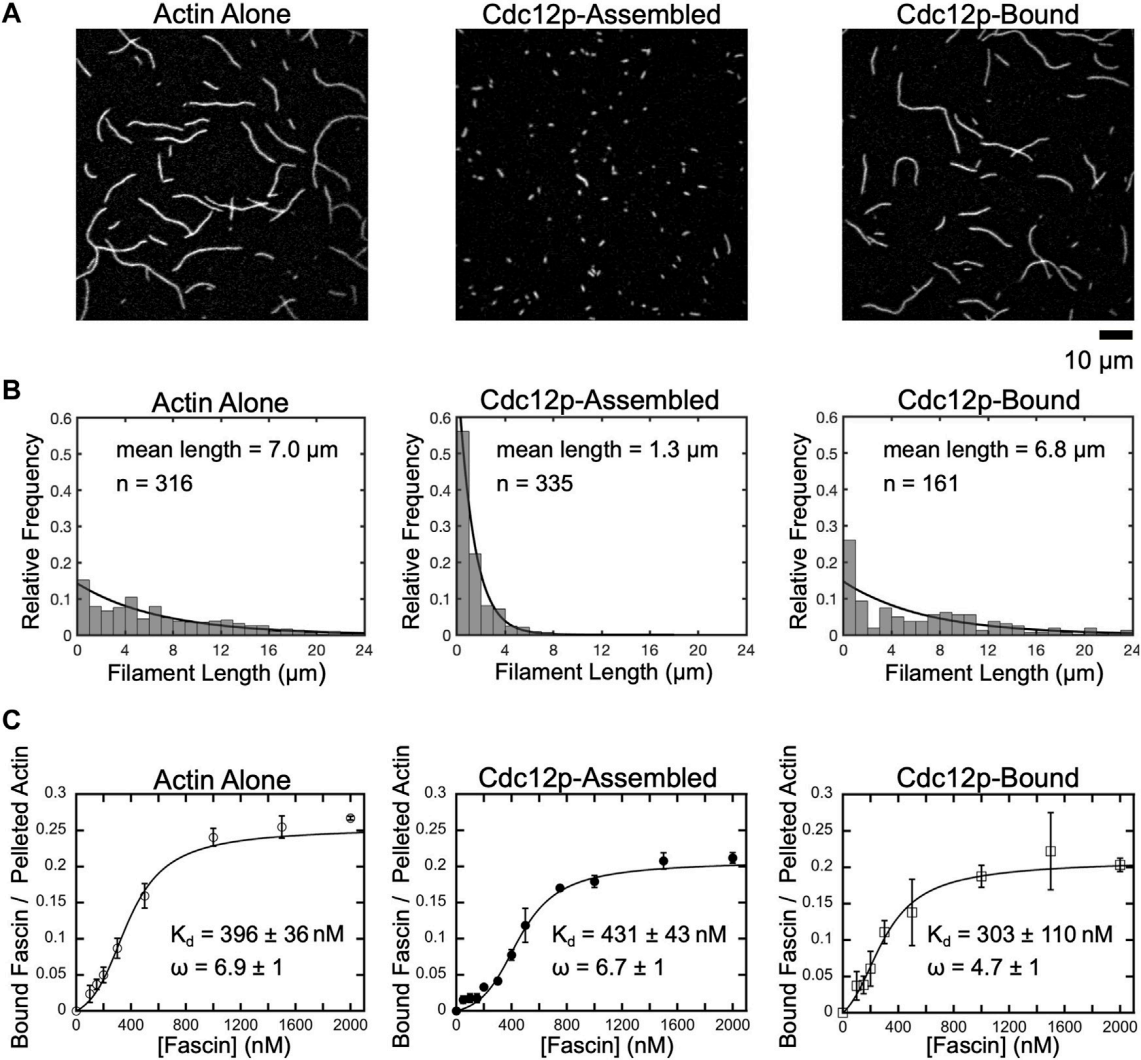
Fascin binds with similar affinity to short and long filaments. **(A)** Representative micrographs of filaments polymerized from 2 µM actin monomers in the absence or presence of 250 nM Cdc12p. Filaments were labeled with FITC-phalloidin, diluted in imaging buffer and visualized by TIRF microscopy. Cdc12p was added prior to polymerization in the “Cdc12p-Assembled” sample and post-polymerization in the “Cdc12p-Bound” sample. **(B)** Histograms of filament lengths measured at equilibrium for reactions polymerized in different conditions. The lines are fits to the data using the exponential equation f_i_ = λexp(-λli). The fitted values for λ are 0.143, 0.769 and 0.147, and the mean filament lengths are 1/λ, or 7.0, 1.3, and 6.8 µm for filaments assembled in reactions containing actin alone (left), filaments assembled in the presence of Cdc12p (middle), and filaments bound by Cdc12p following polymerization, respectively. **(C)** Co-sedimentation assays measuring fascin binding to actin bundles assembled from short and long filaments. Filaments were assembled in the absence or presence of Cdc12p and incubated with a range of fascin concentrations. Reactions were spun at low-speed (10,000 x *g* or 20,000 x *g* for long or short filaments, respectively) to pellet bundles and analyzed by SDS-PAGE. The intensity of the fascin band in the pelleted fraction was divided by the intensity of the actin band in the pelleted (i.e., bundled) fraction for each reaction. Lines are fits of the McGhee-von Hippel cooperative binding model, which yields a binding affinity (K_d_) and cooperativity constant (ω) (McGhee and von Hippel, 1974; De La Cruz, 2005). Error bars are the standard error of the mean values obtained from at least three independent replicates.

To assemble filaments with different lengths, we incubated purified actin monomers in polymerization conditions in the absence and presence of a construct containing the two major actin polymerization domains of Cdc12p (i.e., the FH1 and FH2 domains) (Kovar et al., 2003). Like most formins, Cdc12p nucleates filaments by encircling and stabilizing dimers and trimers of actin monomers with its dimeric FH2 domain (Pring et al., 2003; Moseley et al., 2004; Otomo et al., 2005; Lu et al., 2007; Yamashita et al., 2007). Following nucleation, the FH2 dimer steps onto incoming actin subunits to incorporate them into the filament, thus enabling the formin to remain processively bound at the barbed end of the elongating filament (Courtemanche, 2018). Once each polymerization reaction reached equilibrium, we added fluorescent phalloidin, imaged the filaments using total internal reflection fluorescence (TIRF) microscopy, and quantified their lengths. We found that each reaction robustly assembled into filaments of varying lengths (Figure 1A). The distributions of filaments lengths are well characterized by single exponential fits (Figure 1B), which yield an average filament length and a variance (see Methods) (Sept et al., 1999; Zweifel et al., 2021).

In the absence of formin, we measured an average filament length of 7.0 µm, in agreement with published measurements performed on similar reactions (Sept et al., 1999; Zweifel et al., 2021) (Figure 1B, Actin alone). Addition of Cdc12p following polymerization does not significantly alter the average filament length (Figure 1B, Cdc12p-bound), confirming that formins do not influence the lengths of preassembled actin filaments. In contrast, inclusion of Cdc12p at the time of initiation of polymerization dramatically increases the number of actin filaments assembled over the course of the reaction (Supplementary Figure S1). This increase in filament nucleation is matched by a narrower length distribution that is significantly shifted toward short lengths (1.3 µm average length) (Figure 1B, Cdc12p-assembled).

### Fascin binds with similar affinity to short and long actin filaments

Formin binding has been shown to alter the conformation of the subunits at the barbed ends of actin filaments (Aydin et al., 2018). This structural effect propagates at least 200 nm along the length of the filament and is proposed to influence the association of other actin-binding proteins, including tropomyosin and cofilin (Bugyi et al., 2006; Papp et al., 2006; Skau et al., 2009; Johnson et al., 2014; Mizuno et al., 2018). To determine if binding of Cdc12p to the barbed ends of actin filaments alters fascin’s actin-binding and bundling activities, we performed low-speed co-sedimentation assays with each of our populations of filaments. We found that saturating concentrations of fascin efficiently bundle actin filaments that are polymerized both in the absence and presence of formin (72 ± 3% and 73 ± 3% of the total filamentous actin is incorporated into bundles in the absence and presence of formin, respectively) (Supplementary Figure S2). As previously reported, fascin binds cooperatively to actin filaments with free (i.e., not formin-bound) barbed ends with a dissociation constant of 396 ± 36 nM (Figure 1C, Actin alone) (Jansen et al., 2011; Winkelman et al., 2016). Fascin also binds cooperatively to short and long Cdc12p-bound filaments with similar affinities (K_d_ = 431 ± 43 nM and 303 ± 110 nM), indicating that Cdc12p does not alter fascin’s equilibrium actin-binding or bundling activities (Figure 1C, Cdc12p-assembled, Cdc12p-bound). Binding of phalloidin to actin filaments also does not alter fascin’s binding affinity (Supplementary Figure S3).

### Fascin bundles short and long filaments cooperatively

Fascin’s cooperative interactions with actin filaments indicate that an initial binding event increases the probability of subsequent fascin binding at a neighboring site. This is consistent with the observation that fascin-mediated bundling causes filaments to “zipper” together as binding propagates along the lengths of filaments (Breitsprecher et al., 2011). To determine how filament length influences this cooperative binding process, we visualized fascin-mediated bundling in real-time using TIRF microscopy. Because Cdc12p does not alter fascin’s affinity for actin filaments, we omitted Cdc12p from reactions containing long filaments. The absence of formin in these samples enables filament annealing during sample preparation, which helps to maintain consistent filament length and maximizes differences among samples containing short and long filaments. To mimic a single population of actin filaments at distinct stages of elongation, we used equal concentrations of short and long filaments in our reactions.

**FIGURE 2:**
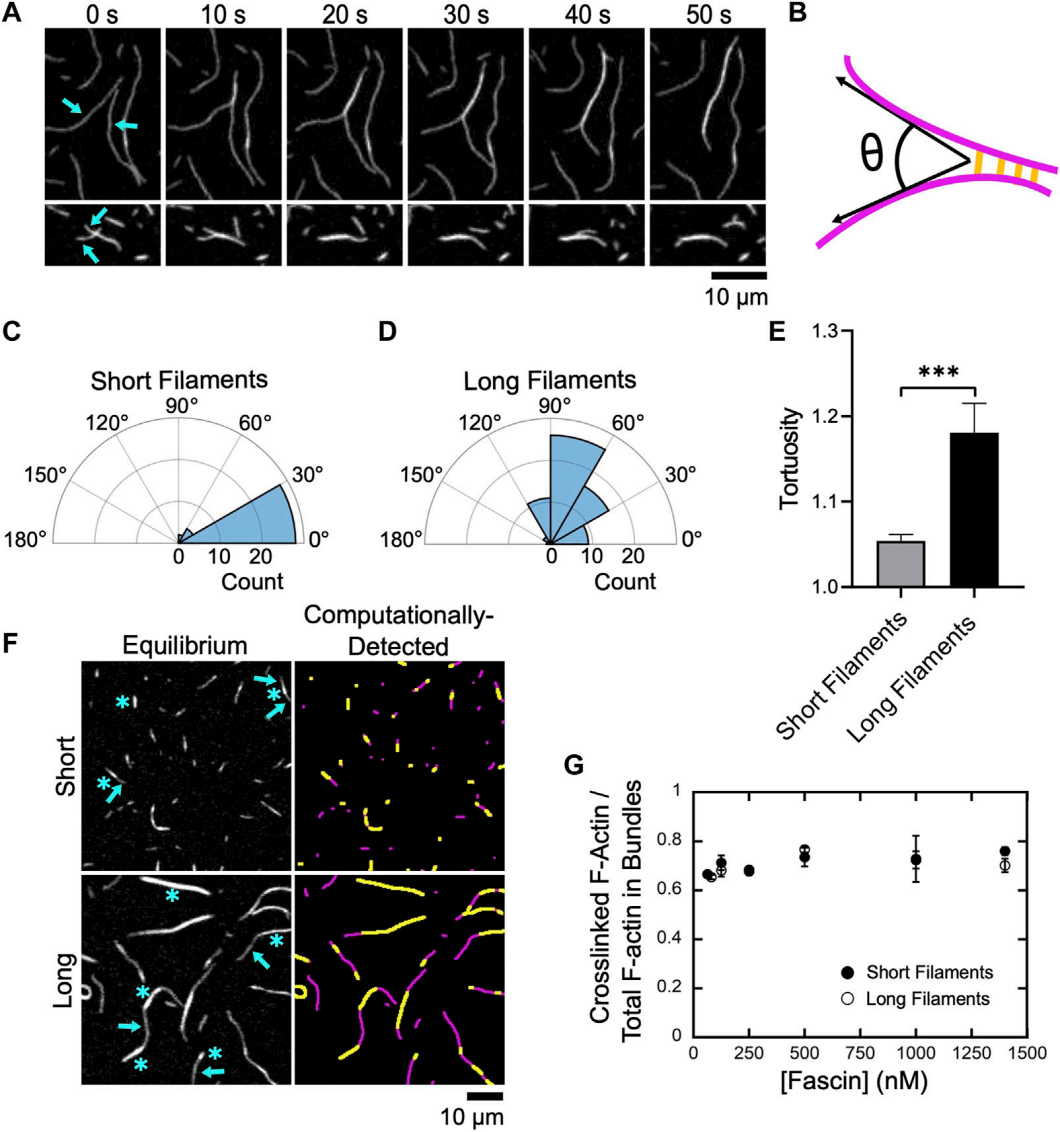
Fascin bundles short and long filaments cooperatively. Filaments were assembled by Cdc12p (short filaments) or via spontaneous actin polymerization (long filaments), labeled with FITC-phalloidin, diluted into microscopy buffer and imaged using TIRF microscopy following the introduction of fascin. **(A)** Representative time series of TIRF micrographs showing bundling of two long filaments (upper panels) and two short filaments (lower panels) in the presence of 1,000 nM fascin. **(B)** Schematic depicting the angle of alignment (θ) defined by two filaments (magenta) as they undergo bundling by fascin (yellow). **(C,D)** Polar histograms of angles of alignment measured for short **(C)** and long filaments **(D)** during the formation of new bundles. Angle measurements were obtained for at least 30 bundling events from three independent replicates. **(E)** Tortuosity (defined as the ratio of the contour length to the end-to-end distance) of actin bundles assembled from short and long filaments (n ≥ 40 bundles from at least three independent replicates). Statistical significance was established using Welch’s *t*-test (*** corresponds to *p* < 0.001). **(F)** Micrographs (left column) and computational detection (right column) of bundles of short and long actin filaments at equilibrium. Bundles contain crosslinked regions (blue asterisks in left panels, yellow stretches in right panels) and stretches of single filaments (blue arrows in left panels, magenta stretches in right panels). **(G)** Dependence of the ratio of the fluorescence intensity of crosslinked regions to the total fluorescence intensity of the filamentous actin contained in bundles on the concentration of fascin for short (closed circles) and long (open circles) filaments. Error bars are the standard error of the mean from at least three independent experiments.

Fascin-mediated bundling requires an initial alignment of two filaments into a parallel orientation and at an inter-filament distance that promotes stable binding of fascin (Courson and Rock, 2010). By observing individual bundling events, we found that alignment often occurs along short stretches of filaments that encounter one another at an angle (Figures 2A,B). By measuring the angles at which filaments encounter one another prior to bundle formation, we found that short filaments must be aligned in a nearly parallel orientation to promote bundling (Figure 2C; average angle of alignment of 14.4°). In contrast, we observed a much larger variation in the relative orientation of long filaments that undergo bundling (Figure 2D; average angle of alignment of 65.5°), consistent with a length-dependent increase in filament flexibility.

Following filament alignment, fascin-mediated bundling produces a quantifiable increase in the fluorescence signal along the lengths of actin filaments (Figure 2A) (Breitsprecher et al., 2011). As each reaction progresses, changes in fluorescence intensity occur along the lengths of crosslinked filaments until bundling is complete and sequential micrographs reveal no further changes.

At equilibrium, bundles assembled from both short and long filaments contain a mixture of crosslinked regions consisting of multiple overlapping filaments, and regions corresponding to stretches of single filaments that contain no crosslinks (Figure 2F, left panels). Bundles also exhibit variable curvature along their lengths. To assay for length-dependent differences in curvature, we measured bundle tortuosity by dividing the contour length of each bundle by its end-to-end distance (Figure 2E). We found that bundles of short filaments exhibit less tortuosity, and are therefore straighter, than bundles of long filaments.

To quantify crosslinking along the lengths of actin bundles, we employed a computational analysis tool developed in-house that uses fluorescence intensity to detect single and overlapping stretches of actin filaments (see Methods) (Figure 2F, right panels). We determined that crosslinked regions comprise ∼70% of the total length of actin bundles at equilibrium (Figure 2G). This structural feature is insensitive to both filament length and fascin concentration, suggesting that this is a general property of actin bundles assembled by fascin and may correspond to the minimum overlap necessary to resist filament separation and bundle disassembly via diffusional forces.

### Fascin-mediated bundling occurs via distinct phases

**FIGURE 3:**
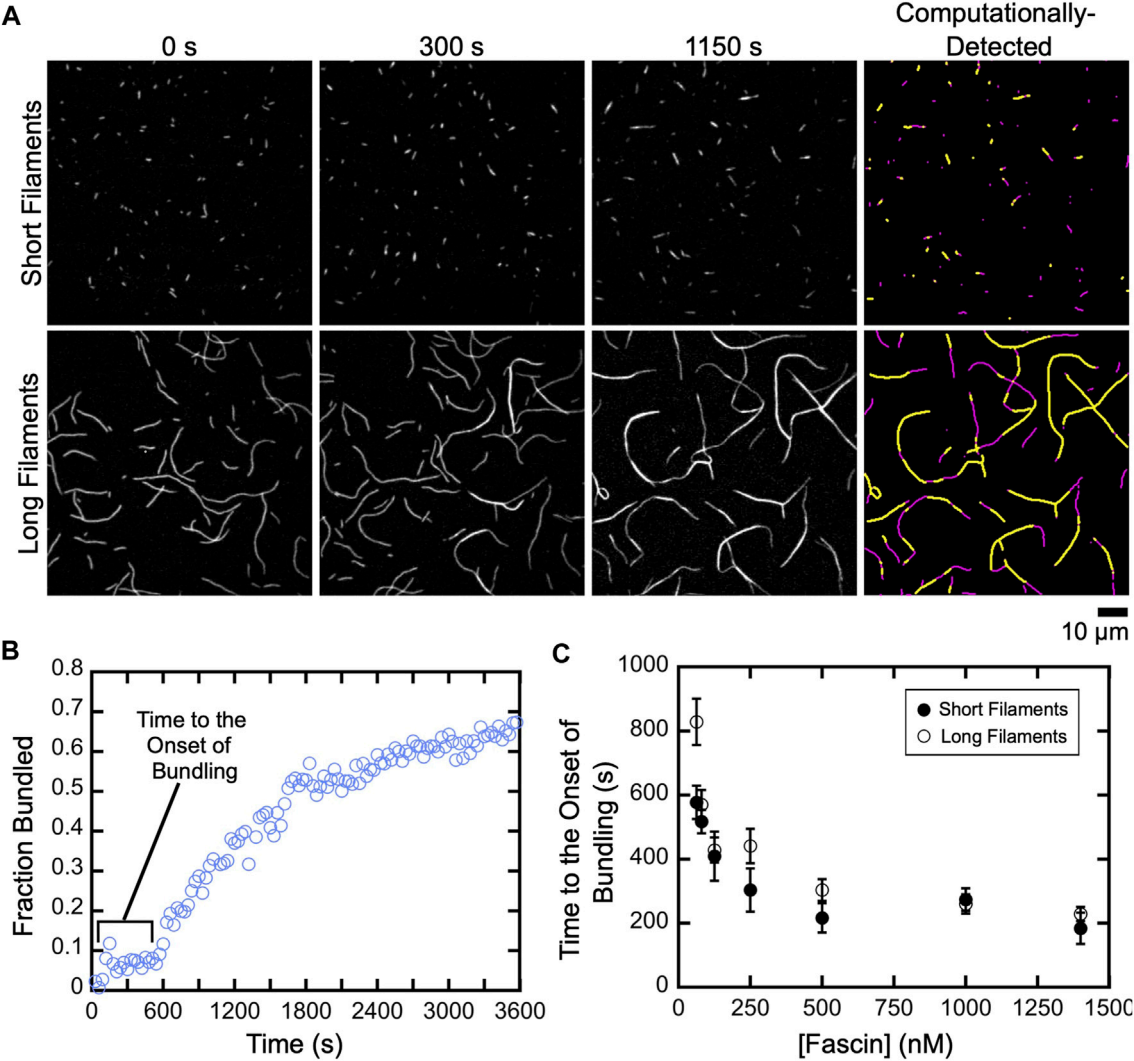
A delay prior to the onset of fascin-mediated bundling is insensitive to filament length. Filaments were assembled in the absence or presence of Cdc12p, labeled with FITC-phalloidin and visualized by TIRF microscopy following the addition of a range of concentrations of fascin. **(A)** Micrographs collected at various time points to show the progress of representative bundling reactions containing 1,400 nM fascin. Panels on the right show automated detection of stretches of bundled (yellow) and single (magenta) filaments at 1,150 s. **(B)** The fraction of the filamentous actin that is bundled over time for a representative reaction containing long filaments and 80 nM fascin. **(C)** Dependence of the length of the delay prior to the onset of bundling on the concentration of fascin for reactions containing short (closed circles) and long (open circles) filaments. Error bars are the standard error of the mean of three independent replicates.

To investigate the effects of filament length on the rate of bundle assembly, we quantified the fraction of filamentous actin that is crosslinked over the course of reactions containing either short or long filaments (Figures 3A,B; Supplementary Movies S1, 2). For both populations, we observed a delay between the addition of fascin and the onset of bundling. The length of this delay decreases as the fascin concentration increases, until a plateau is reached at 500 nM fascin (Figure 3C). This indicates that the initiation of bundling depends on the rate at which fascin binds actin. Increasing the concentration of actin also decreases the length of the delay, consistent with a dependence on the rate of diffusional encounters between filaments (Supplemental Figure S4). Despite the dependence on actin concentration, the delay is insensitive to filament length, except at the lowest sampled fascin concentration, suggesting that filament length does not influence the rate at which fascin binding is initiated.

**FIGURE 4:**
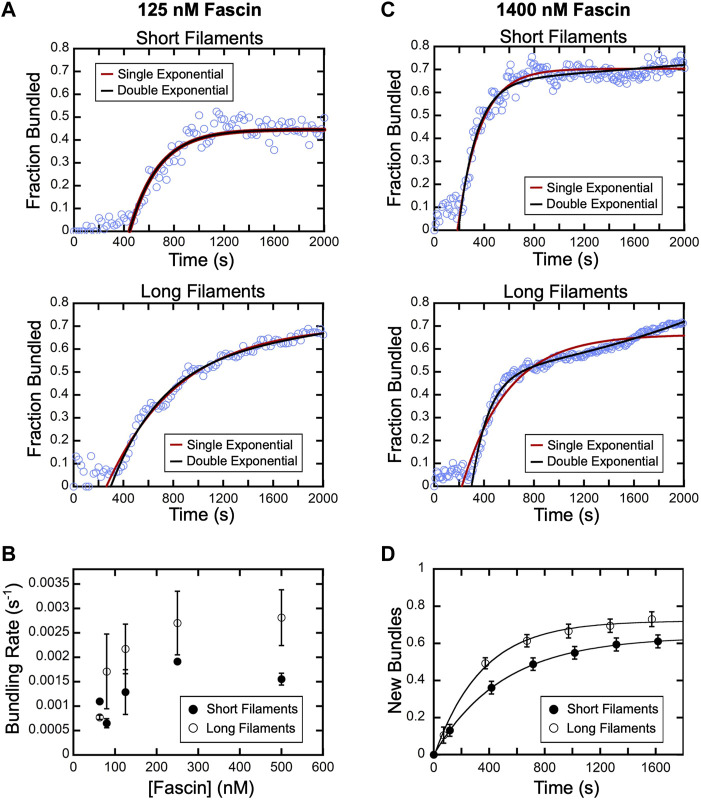
Fascin-mediated bundling occurs via distinct phases. A range of concentrations of fascin was introduced into reactions containing FITC-phalloidin-labeled actin filaments. **(A)** The fraction of the filamentous actin that is bundled over time in representative reactions containing 125 nM fascin and short (top) or long (bottom) filaments. Red and black lines are single and double exponential fits to the data. **(B)** Bundling rates for short (closed circles) and long (open circles) actin filaments obtained from single exponential fits to bundling profiles collected at sub-saturating fascin concentrations. Error bars are the standard error of the mean bundling rate measured in at least three independent experiments. **(C)** The fraction of the filamentous actin that is bundled over time in representative reactions containing 1,400 nM fascin and short (top) or long (bottom) filaments. Red and black lines are single and double exponential fits to the data. **(D)** New bundles assembled from short (closed circles) and long filaments (open circles) over time in the presence of 1,400 nM fascin. To facilitate comparison, the number of bundles that are assembled *de novo* over time was normalized to the number of filaments present at the beginning of each reaction. Lines are single exponential fits to the data, which yield rates of new bundle formation of 0.002 s^−1^ and 0.003 s^−1^ for reactions containing short and long filaments, respectively. Error bars are the standard error of the mean number of bundles measured in at least three independent experiments.

Following the initial delay, the fraction of actin that is bundled increases over time until a plateau is reached (Figure 3B). Bundling profiles collected at fascin concentrations up to 500 nM (which corresponds to ∼60% saturation of the available binding sites) are well described by single exponential functions, independent of filament length (Figure 4A). This is consistent with a single kinetic bundling phase at these concentrations of fascin. The rate constants generated by fits to the data reveal that reactions containing short filaments are bundled more slowly than reactions containing long filaments (Figure 4B). Bundling rates measured in reactions containing long filaments are more variable than those obtained with short filaments. The magnitude of this variability is directly proportional to the variance in the lengths of the filaments in our populations of short and long filaments. Despite the increased variability observed in reactions containing long filaments, the differences between the bundling rates for short and long filaments are statistically significant across all fascin concentrations (Welch’s *t*-test; *p* < 0.03), except for the lowest sampled concentration.

At concentrations exceeding 500 nM fascin, bundling of short filaments remains well described by a single exponential function (Figure 4C). However, fitting bundling profiles for long filaments requires a double exponential function, revealing the emergence of a second kinetic bundling phase. The relative amplitudes of the two kinetic phases correspond to approximately 90% and 10% of the total amplitude of the bundling reaction. Therefore, one of the phases makes a minor contribution to the fraction of filamentous actin that is incorporated into bundles over the course of the reaction.

### An increase in filament length promotes bundle expansion

Bulk bundling reactions contain numerous filaments that progress through bundle assembly at different times (Figure 3A; Supplementary Movies S1, 2). This asynchrony can lead to temporal overlap of otherwise discrete bundling phases, complicating their resolution. To determine the origin of the two bundling phases in our reactions with long filaments, we therefore examined the different types of bundling events that take place in the presence of a saturating concentration of fascin (i.e., 1,400 nM fascin). We first quantified the rate of *de novo* bundle formation in our reactions. Formation of new bundles involves the crosslinking of two individual filaments together and excludes bundle “expansion” events, in which pre-existing bundles incorporate additional filaments. Consistent with our bulk bundling measurements (Figure 3B), we found that the number of new bundles increases over the course of the reaction (Figure 4D). Fitting the data with single exponential functions reveals that new bundle formation is slower for short filaments than for long filaments (0.002 s^−1^ and 0.003 s^−1^, respectively). The assembly of new bundles in reactions containing long filaments is completed within ∼1,200 s. At this time point, ∼60% of the filamentous actin has been incorporated into bundles, which corresponds to 85% of the total amplitude of the bundling reaction (Figure 4C, Long filaments). This suggests that the formation of new bundles dominates the first phase of bundle assembly.

**FIGURE 5:**
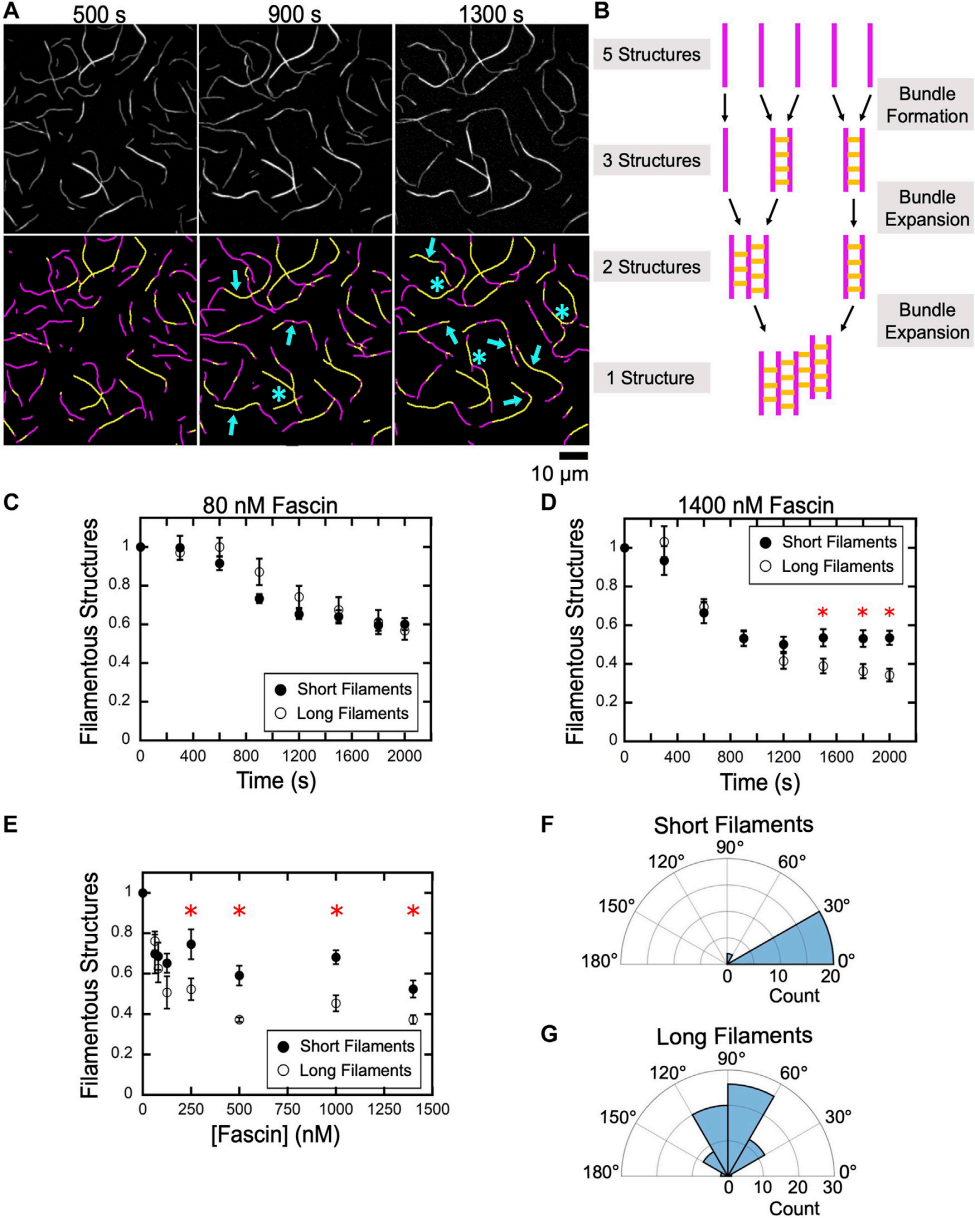
An increase in filament length promotes bundle expansion. Actin filaments were labeled by FITC-phalloidin, bundled by a range of concentrations of fascin and imaged by TIRF microscopy. **(A)** *Top row,* TIRF Micrographs collected at 400 s intervals spanning the first and second phases of a representative bundling reaction containing long filaments and 1,400 nM fascin. *Bottom row,* Computationally-detected stretches of bundled (yellow) and single (magenta) filaments. Blue arrows indicate regions where a pre-existing bundle has expanded by incorporating an additional filament or merging with another bundle. Blue asterisks indicate nonlinear bundle morphologies. **(B)** Schematic illustrating the relationship between bundle assembly progression and the number of filamentous structures contained in a bundling reaction. Filaments are depicted in magenta. Fascin-mediated crosslinks are shown in yellow. **(C–E)** The number of filamentous structures (i.e., the sum of the individual filaments and bundles) visualized over time for reactions containing 80 nM **(C)** and 1,400 nM **(D)** fascin. The number of bundles was normalized to the number of filaments present at the beginning of each reaction. Red asterisks indicate statistically significant differences (*p* < 0.05 using Welch’s *t*-test) between measurements obtained for reactions containing short and long filaments. Error bars are the standard error of the mean number of filamentous structures measured in at least three independent experiments. **(E)** The dependence of the number of filamentous structures visualized in bundling reactions that have reached equilibrium on the concentration of fascin. **(F,G)** Polar histograms of angles of alignment for short **(F)** and long **(G)** filaments across three independent experiments.

In reactions containing 1,400 nM fascin, bundling of long filaments continues after the assembly of new bundles is complete (Figure 4C, Long filaments). To determine the mechanism driving this continuation of the bundling reaction, we compared micrographs collected at various time points over the course of the first and second exponential phases (Figure 5A). We found that bundling that occurs early in the first phase (∼500 s) is characterized by the formation of new bundles. These early bundles tend to have linear morphologies (defined by the presence of two bundle ends) and a single continuous crosslinked region. Toward the end of the first phase (∼900 s), most of the single actin filaments have been incorporated into bundles. In contrast, bundles observed during the second phase often contain more than two filaments, multiple crosslinked regions, and branched morphologies (defined by the presence of more than two bundle ends), suggesting that discrete bundles become interconnected by merging together during the second phase (Figure 5A, arrows, asterisks).

When individual filaments become crosslinked into bundles, the number of discrete filamentous structures contained in the reaction decreases. This number further decreases as bundles expand by incorporating additional filaments or coalesce with other preassembled bundles (Figure 5B). To assess the contributions of bundle expansion to the kinetics of bulk bundling reactions, we measured the number of filamentous structures (i.e., the sum of the individual actin filaments and filament bundles) present in our reactions as bundling progresses. In reactions that undergo bundling in a single kinetic phase, we found that the number of filamentous structures decreases as the reaction proceeds and reaches a plateau at approximately 60% of the initial number of structures (Figure 5C). The magnitude of this change is similar in samples containing short and long filaments, indicating that, at equilibrium, each filamentous structure contains approximately two filaments. This is consistent with the absence of a distinct bundle expansion phase.

In the presence of 1,400 nM fascin, the number of filamentous structures observed in reactions containing short and long filaments decreases at similar rates and with the same magnitude until the end of the first phase (∼1,000 s) (Figure 5D). At longer times, the number of structures remains unchanged for short filaments but continues to decrease until a plateau is reached at approximately 30% of the initial number of structures in reactions containing long filaments. This value indicates that the assembled bundles contain more than two filaments on average, thus supporting a model for bundle expansion in reactions containing long filaments.

To assess the effects of bundle expansion on the overall morphology of bundled actin networks, we quantified the number of filamentous structures in reactions that have reached equilibrium as a function of the concentration of fascin (Figure 5E). We found that bundling decreases the number of filamentous structures by 30–50% in reactions containing short filaments, corresponding to an average of two or fewer actin filaments per structure. Reactions containing long filaments assemble into a similar number of filamentous structures at concentrations of fascin below 150 nM. At higher fascin concentrations, long filaments assemble into a smaller number of structures than do short filaments.

Despite the absence of a distinct bundle expansion phase, bundles of short filaments occasionally incorporate additional filaments or merge with other bundles over the course of our reactions. These expansion events require the alignment of merging bundles into a nearly parallel orientation (Figure 5F; average angle of alignment of 16.8°). As a result, short filament bundles retain linear morphologies following expansion. In contrast, bundles of long filaments can form inter-bundle crosslinks when oriented at a wide range of angles, thereby promoting the assembly of branched bundles (Figure 5G; average angle of alignment of 86.6°). Taken together, our results indicate that fascin crosslinks short filaments into bundles that remain linear and discrete over the course of the reaction, whereas bundles of long filaments coalesce into interconnected networks with irregular morphologies.

### Bundles of short filaments remain discrete while elongating

The results of our experiments with short and long actin filaments suggest that filament length plays a central role in regulating bundle assembly by dictating the probability of forming interconnections between bundles. To determine how the length at which filaments initially become crosslinked impacts the architecture of dynamic actin networks, we investigated whether filament elongation can alter the organization of preassembled actin bundles. We introduced actin monomers and fascin into reactions containing bundles of short, Cdc12p-bound filaments and visualized elongation using TIRF microscopy. To promote Cdc12p-mediated filament elongation and inhibit spontaneous filament nucleation, we included 10 µM profilin in each reaction. Profilin is a cytoplasmic protein that binds actin monomers and sterically hinders the inter-monomer associations that are necessary for filament nucleation (Pollard and Cooper, 1984). Binding of profilin-actin complexes to polyproline tracts located in formin FH1 domains promotes their delivery to the barbed end, where they are incorporated into the filament via stepping of the FH2 domain (Courtemanche, 2018).

**FIGURE 6:**
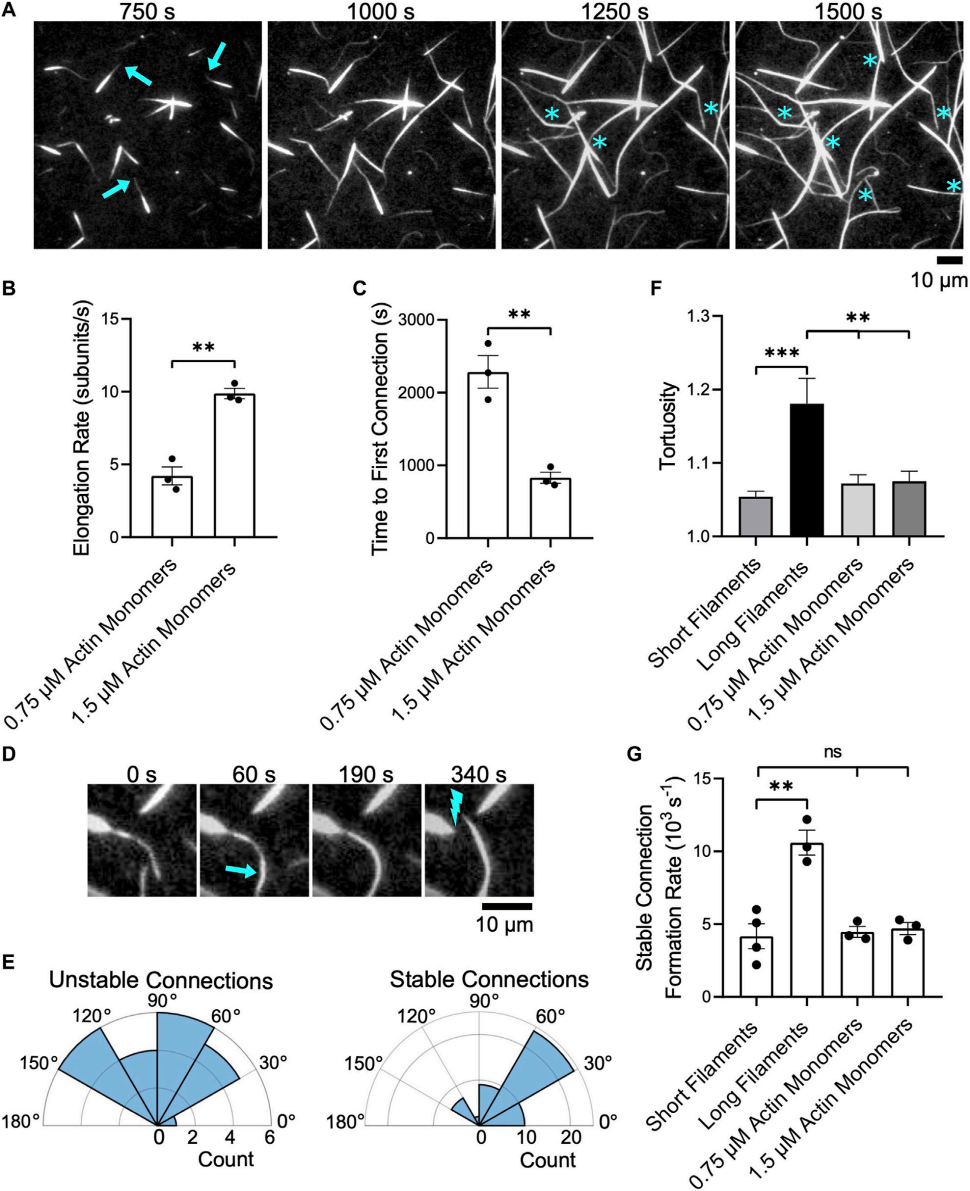
Bundles of short filaments remain discrete while elongating. Filaments were assembled in the presence of Cdc12p, labeled with FITC-phalloidin and bundled by 1 µM fascin. Bundles were visualized by TIRF microscopy following the introduction of 0.75 or 1.5 µM actin monomers (5% Oregon Green-labeled), 10 µM *S. pombe* profilin, and 1 µM additional fascin. In all plots, asterisks indicate statistical significance (** indicates *p* < 0.01; *** indicates *p* < 0.001; “n.s.” indicates *p* > 0.05). Black dots represent values from individual replicates. Error bars are the standard error of the mean measurements obtained from three independent experiments. **(A)** Time series of micrographs of preassembled bundles elongating following the addition of 1.5 µM actin monomers (5% Oregon Green-labeled). Arrows indicate growing barbed ends. Blue asterisks denote crosslinking events that connect two pre-existing bundles. **(B)** Rates of bundle elongation measured in the presence of 0.75 and 1.5 µM actin monomers. **(C)** Length of time required to establish the first connection between bundles following the onset of elongation. **(D)** Micrographs showing an example of two elongating bundles forming a short-lived connection. The arrow indicates a newly crosslinked region and the lightning bolt indicates the site of subsequent bundle breakage. **(E)** Polar histograms showing the distribution of the angles of alignment for unstable (left) and stable (right) connections formed between preassembled bundles elongating in the presence of 1.5 µM actin monomers. **(F)** Tortuosity (defined as the ratio of the contour length to the end-to-end distance) of bundles assembled from filaments of constant length or elongating actin bundles (n ≥ 40 bundles from at least three independent experiments). **(G)** Rates at which stable inter-bundle connections are formed in reactions containing filaments of constant length or elongating bundles. These rates were measured by counting the number of instances in which bundles incorporate additional filaments or coalesce with other bundles without undergoing subsequent breakage over the time course of the reaction.

Following the addition of actin monomers and profilin, preassembled bundles elongate at only one end (Figure 6A, arrows), reflecting the parallel orientation of filaments bundled by fascin*.* The growing ends of bundles appear dimmer than the rest of the bundle owing to the lower fluorescence intensity of filaments assembled by formins from Oregon green-labeled monomers and profilin (Sherer et al., 2018) compared to FITC-phalloidin-labeled filaments. This feature facilitates the identification of each bundle’s barbed end and enables us to track the relative orientation of each bundle over time. Preassembled bundles elongate approximately half as fast as single filaments in identical polymerization conditions (Figure 6B) (Zweifel and Courtemanche, 2020), consistent with published reports of the inhibitory effects of bundling on polymerization (Suzuki et al., 2020).

As we observed in reactions containing single filaments, we found that elongating bundles occasionally establish interconnections with other bundles (Figure 6A, asterisks). Doubling the concentration of actin monomers doubles the elongation rate and decreases the length of the delay between the initiation of bundle elongation and the formation of the first inter-bundle connection by ∼50% (Figures 6B,C). Following the formation of the first inter-bundle connection, elongating bundles continue to merge with one another until equilibrium is reached. The rate at which these inter-bundle connections form increases with the filament elongation rate (Supplementary Figure S5A). However, many of these connections are transient and break as elongation and crosslinking progress (Figure 6D). These “unstable” connections form between elongating bundles that are oriented at a wide range of angles (Figure 6E, Unstable connections). To distinguish transient connections from long-lived binding events that ultimately alter the architecture of the bundled actin network, we classify connections that persist without breaking over the course of our bundling reactions as “stable”. Whereas 95% of the inter-bundle connections that form in reactions containing non-elongating filaments are stable, only 69% of the connections formed between elongating preassembled bundles are stable. These long-lived connections are established between elongating bundles that are oriented into a relatively narrow range of angles (Figure 6E, Stable connections). These angles are more acute, and are therefore closer to a parallel orientation, than those observed during the expansion phase in reactions containing long single filaments (Figure 5G). As a result, the establishment of stable connections gives rise to networks containing large, straight bundles that exhibit less curvature than bundles formed in reactions containing individual, long filaments (Figure 6F).

The rate at which unstable, transient connections form increases with the filament elongation rate (Supplementary Figure S5B). In contrast, the rate at which stable inter-bundle connections form is insensitive to the rate of elongation and matches the rate at which non-elongating, short bundles form inter-bundle connections (Figure 6G). This rate is approximately 60% slower than the rate at which long filament bundles form inter-bundle connections. As a result, reactions containing actin bundles that are preassembled from short filaments contain a larger number of discrete bundles than do reactions containing long filaments, despite attaining similar filament lengths via elongation.

## Discussion

The construction of higher-order actin structures requires the polymerization and crosslinking of actin filaments into networks with specific architectures (Blanchoin et al., 2014). To understand how the process of filament elongation regulates the dynamic assembly of polarized actin bundles, we examined the effects of filament length on fascin-mediated bundling. We found that filament length directly influences both the rate of bundle formation and the likelihood that discrete bundles will expand and merge to form interconnected networks.

### A model for length-dependent filament bundling

**FIGURE 7:**
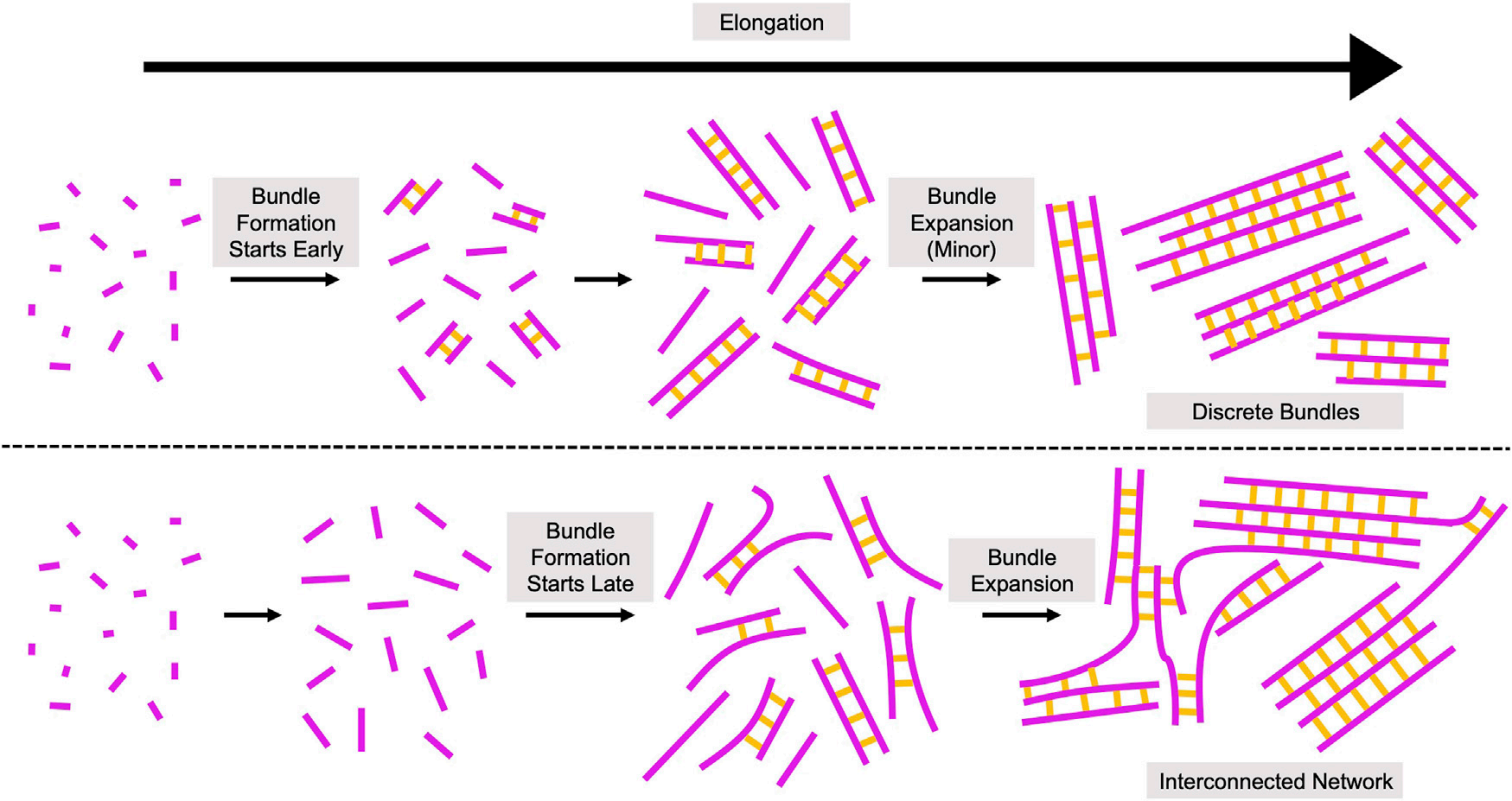
Assembly of Crosslinked Actin Structures from Elongating Filaments. Proposed model for the role of filament length in the assembly of actin bundles. Elongating actin filaments and fascin-mediated crosslinks are depicted in magenta and yellow, respectively. Bundling occurs via two phases: bundle formation and bundle expansion. During bundle formation, individual filaments become crosslinked together to form new bundles. During bundle expansion, pre-existing bundles incorporate additional filaments and merge with other bundles. Initiation of filament bundling early in elongation (when filaments are short) promotes the assembly of topologically discrete bundles (top). Initiation of bundling at later stages of elongation (when filaments are longer) produces interconnected networks with irregular bundle morphologies (bottom).

Based on our quantifications of filament bundling reactions, we propose that bundling occurs via two distinct phases (Figure 7). During the first phase, individual filaments become crosslinked together to form new bundles. During the second phase, pre-existing bundles expand by incorporating additional filaments and merging with other bundles. Our data suggest that the bundle formation phase dominates reactions containing either short filaments or sub-saturating concentrations of fascin (Figures 4, 5). The rate of bundle formation increases with filament length, suggesting that the rate of filament elongation might regulate this process. The limited contribution of bundle expansion to these reactions enables the formation of discrete bundles that contain few filaments. In contrast, bundles assembled from long filaments undergo significant expansion by incorporating additional filaments and merging with one another in the presence of fascin concentrations exceeding 250 nM (corresponding to >25% saturation of the available fascin binding sites). This process leads to bundle thickening and shifts the architecture of the bundles into an interconnected network. By comparing the number of filamentous structures prior to and following bundling, we found that reactions containing long filaments undergo bundle expansion in the presence of 250–500 nM fascin despite the absence of a distinct second kinetic bundling phase. This suggests that bundle formation and expansion progress at similar rates in these reactions, in contrast to the relatively fast rates of bundle formation and slower rates of bundle expansion we observe at saturating fascin concentrations.

Taken together, our results highlight an important regulatory role for polymerization-promoting proteins like formins and Ena/VASP proteins (Pollard, 2016). Whereas filaments that elongate slowly are likely to become crosslinked while they are still relatively short, filaments that elongate rapidly are more likely to attain longer lengths before becoming crosslinked. By mediating changes in filament length, polymerization-promoting proteins can therefore directly impact both the rate of bundle assembly and the architecture of the resultant bundled network.

In addition to the elongation rate, the persistence length of actin filaments (10 µm (Isambert et al., 1995; McCullough et al., 2008)) is likely a key determinant of bundling propensity. Whereas short filaments are relatively straight, long filaments are more likely to exhibit curvature and thus sample a range of conformations. This increased flexibility facilitates the alignment of stretches of filaments into orientations that are compatible for bundling (Figure 2D), thereby speeding bundle formation (Figure 4B). Following initial bundle assembly, incorporation of additional filaments into pre-existing bundles depends on the probability that two filamentous structures will become aligned in an orientation that is compatible for bundling. This probability increases with filament length as the range of angles into which filamentous structures can be oriented to promote bundling also increases (Figures 5F,G).

We found that stretches of single filaments lacking crosslinks are typically located at bundle ends and comprise approximately 30% of the total length of each bundle (Figure 2G). As a result, bundles of long filaments possess longer stretches of single filaments than bundles of short filaments. As with bundle formation, the length of these regions likely dictates the probability that they will contact other filaments or bundles at orientations that are compatible for fascin binding. Thus, longer stretches of single filaments promote bundle expansion. On the other hand, the presence of short stretches of single filaments indicates that the barbed ends of the bundled filaments are in relatively close spatial proximity to one another. This alignment of the filaments likely imparts uniform structural rigidity along the length of elongating bundles, thus enabling protrusive structures to withstand compression as they deform the plasma membrane and assemble outward from the cell body.

### Filament length at the onset of bundling influences the architecture of bundled actin networks

To determine whether the lengths at which filaments initially become crosslinked constrains the architecture of dynamically elongating networks, we visualized changes in the morphologies of preassembled bundles of Cdc12p-bound short filaments under polymerization conditions. We found that bundles elongate unidirectionally and occasionally become crosslinked with neighboring bundles. We determined that the length of time required to establish the first inter-bundle connection is inversely dependent on the elongation rate. Following this initial inter-bundle crosslinking event, subsequent connections form among other elongating bundles. The rate at which these connections are formed also positively correlates with the elongation rate but is slower than the rate measured in reactions containing long single filaments (Supplementary Figure S5A).

Approximately one third of the connections formed between elongating bundles are short-lived and are followed by filament breakage at or near the initial site of crosslinking (Figure 6D). Since fascin is an inflexible crosslinker that assembles rigid bundles (Claessens et al., 2006; Van Audenhove et al., 2016), the breakage of these short-lived connections is likely caused by geometric constraints imposed by fascin-mediated crosslinking. Based on our observations, we classified connections that persist throughout our reactions as “stable.” These connections are formed following the alignment of bundles into a narrow range of acute angles, which promotes the retention of bundle linearity following their coalescence. Thus, although filament elongation enables the formation of inter-bundle connections, our data suggest that crosslinking early in elongation pre-aligns growing filaments, setting a template for continued bundle assembly as elongation proceeds. This initial alignment constrains the flexibility of the bundled filaments, increases their resistance to large changes in curvature and inhibits their coalescence into interconnected networks with branched morphologies. As a result, preassembled bundles of short filaments remain straighter following elongation than bundles assembled from long filaments of the same length (Figure 6F).

### Physiological implications of a length-dependent mechanism that regulates the architecture of crosslinked actin networks

The specialized architectures of higher-order actin structures dictate their rigidity, mechanosensitivity, contractility, and lifetime (Reymann et al., 2012; Gressin et al., 2015; Ennomani et al., 2016; Chugh et al., 2017; Stam et al., 2017; Ajeti et al., 2019). These physical properties enable actin structures to perform specific biological functions and must therefore be tightly regulated. We have found that uncoordinated filament elongation and crosslinking can alter the architecture of bundled actin networks assembled by fascin, thus highlighting the importance of filament length regulation during the assembly of protrusive actin structures.

In cells, a large fraction of the filaments that are incorporated into bundled actin structures are polymerized by formins (Schirenbeck et al., 2005; Lizárraga et al., 2009; Mellor, 2010; Jaiswal et al., 2013). Most eukaryotes express at least two formin isoforms, which nucleate and direct the elongation of actin filaments at a wide range of isoform-specific rates (Kovar et al., 2006; Schönichen and Geyer, 2010; Pruyne, 2016). Our results suggest that the elongation properties of formin isoforms likely play a central role in the precise incorporation of actin filaments into structures with specific architectures by controlling the rate of change in filament length. Similarly, the association rate and structural properties of the crosslinking protein are also determinants of actin network architecture (Bartles, 2000; Winkelman et al., 2016; Svitkina, 2018). For example, flexible crosslinkers like α-actinin and filamin A are less sensitive to the relative alignment and orientations of actin filaments than are rigid crosslinkers like fascin (Popowicz et al., 2006; Sjöblom et al., 2008; Courson and Rock, 2010). These crosslinkers may therefore facilitate the formation of a larger number of stable connections among elongating bundles than we observed using fascin. This would enable filament elongation to transform disconnected bundles into interlinked networks.

Perturbation of the expression levels of regulators of actin filament length has been shown to disrupt the assembly and functions of several bundled actin structures in cells. For example, depletion of the formin Dia1 decreases the thickness of the actin cortex in HeLa cells (Chugh et al., 2017). In contrast, depletion of capping protein, which binds and inhibits the elongation of actin filaments, increases actin cortex thickness (Chugh et al., 2017). In both cases, cortical actin tension decreases during mitosis, indicating that an optimal cortical network architecture is achieved through crosslinking of filaments of intermediate lengths. In budding yeast, deletion of the myosin passenger protein Smy1p, which binds and slows polymerization mediated by the formin Bnr1p, results in an increase in actin cable length and curvature (Chesarone-Cataldo et al., 2011). Simulations of actin cable assembly have revealed that this phenotype arises from an increase in inter-cable crosslinking that occurs when the rate of filament polymerization is abnormally fast (Tang et al., 2015). Our results support this model by indicating that the probability that discrete bundles of actin filaments will assemble into interconnected networks increases with filament length.

In fission yeast, cytokinetic contractile ring assembly proceeds via a “search-capture-pull” mechanism in which formins localize to large protein assemblies called “nodes” and polymerize actin filaments (Vavylonis et al., 2008). Myosin II in neighboring nodes binds and pulls on the actin filaments, leading to the formation of a uniform, contractile actomyosin ring. Disruption of cofilin-mediated actin filament severing produces an abnormal clumping of nodes during cytokinetic ring assembly (Chen and Pollard, 2011). This phenotype is similar to the defects observed when the number of long-lived inter-filament connections is increased in simulations of contractile ring assembly. Thus, filament length regulation through severing is required to disassemble improper inter-filament crosslinks that otherwise disrupt the architectural integrity of contractile rings.

Modulating the expression levels and activities of crosslinkers also leads to anomalies in actin structure formation and function. For example, cells expressing a mutant version of fimbrin (Sac6p) that has a weakened affinity for actin filaments assemble shorter and fewer actin cables in budding yeast despite normal rates of filament elongation by formins (Miao et al., 2016). This phenotype may arise from a delay in fimbrin-mediated crosslinking, which would lead to the formation of aberrant inter-filament connections and the assembly of meshworks of long actin filaments rather than the discrete bundles that are characteristic of wildtype actin cables. Similarly, whereas phosphorylation of fascin at serine 39 decreases its bundling activity (Yamakita et al., 1996; Kliewe et al., 2017), expression of a fascin variant that cannot be phosphorylated (S39A) leads to the hyperaccumulation of actin bundles near the cortex in LLC-PK1 cells (Adams et al., 1999). These cells also exhibit reduced spreading on fibronectin-coated surfaces. Our results support a model in which constitutive activation increases the likelihood that fascin will bind short actin filaments, thus spatially aligning their barbed ends at an early stage and promoting the assembly of mechanically robust bundles as polymerization continues.

RNAi-induced silencing of fascin decreases the number of filopodia that are assembled in mouse melanoma cells (Vignjevic et al., 2006; Jaiswal et al., 2013). These filopodia exhibit a “wavy” morphology, suggesting a decrease in their mechanical stiffness (Vignjevic et al., 2006; Jaiswal et al., 2013). Similarly, knockdown of fascin in the human melanoma cell line CHL-1 decreases the number, length, and lifetime of invadopodia and reduces the matrix degradation activity of these protrusions (Li et al., 2010). In both cases, limited fascin availability may delay filament crosslinking and decrease the stiffness of growing actin bundles, causing filament elongation to stall. In this way, disruption of the interdependent kinetics of filament elongation and crosslinking during the assembly of filopodia and invadopodia may provide a molecular mechanism by which downregulation of fascin expression decreases the probability of cancer metastasis (Weaver, 2006; Jacquemet et al., 2015). On the other hand, the high fascin expression levels that are often associated with aggressive metastatic cancers (Hashimoto et al., 2005) may guarantee filament crosslinking early in elongation, thus promoting the robust assembly of invasive actin protrusions.

## Data Availability

The original contributions presented in the study are included in the article/Supplementary Material further inquiries can be directed to the corresponding author.

## References

[B1] Adams J. C. Clelland J. D. Collett G. D. M. Matsumura F. Yamashiro S. Zhang L. (1999). Cell-matrix adhesions differentially regulate fascin phosphorylation. Mol. Biol. Cell 10, 4177–4190. 10.1091/mbc.10.12.4177 10588651 PMC25751

[B2] Ajeti V. Tabatabai A. P. Fleszar A. J. Staddon M. F. Seara D. S. Suarez C. (2019). Wound healing coordinates actin architectures to regulate mechanical work. Nat. Phys. 15, 696–705. 10.1038/s41567-019-0485-9 31897085 PMC6939997

[B3] Andrianantoandro E. Pollard T. D. (2006). Mechanism of actin filament turnover by severing and nucleation at different concentrations of ADF/cofilin. Mol. Cell 24, 13–23. 10.1016/j.molcel.2006.08.006 17018289

[B4] Arthur A. L. Crawford A. Houdusse A. Titus M. A. (2021). VASP-mediated actin dynamics activate and recruit a filopodia myosin. eLife 10, e68082. 10.7554/eLife.68082 34042588 PMC8352590

[B5] Aydin F. Courtemanche N. Pollard T. D. Voth G. A. (2018). Gating mechanisms during actin filament elongation by formins. eLife 7, e37342. 10.7554/eLife.37342 30035712 PMC6056239

[B6] Bartles J. R. (2000). Parallel actin bundles and their multiple actin-bundling proteins. Curr. Opin. Cell Biol. 12, 72–78. 10.1016/S0955-0674(99)00059-9 10679353 PMC2853926

[B7] Blanchoin L. Boujemaa-Paterski R. Sykes C. Plastino J. (2014). Actin dynamics, architecture, and mechanics in cell motility. Physiol. Rev. 94, 235–263. 10.1152/physrev.00018.2013 24382887

[B8] Breitsprecher D. Kiesewetter A. K. Linkner J. Urbanke C. Resch G. P. Small J. V. (2008). Clustering of VASP actively drives processive, WH2 domain-mediated actin filament elongation. EMBO J. 27, 2943–2954. 10.1038/emboj.2008.211 18923426 PMC2585163

[B9] Breitsprecher D. Koestler S. A. Chizhov I. Nemethova M. Mueller J. Goode B. L. (2011). Cofilin cooperates with fascin to disassemble filopodial actin filaments. J. Cell Sci. 124, 3305–3318. 10.1242/jcs.086934 21940796 PMC4074248

[B10] Bryan J. Kane R. E. (1978). Separation and interaction of the major components of sea urchin actin gel. J. Mol. Biol. 125, 207–224. 10.1016/0022-2836(78)90345-5 731692

[B11] Bugyi B. Papp G. Hild G. Lõrinczy D. Nevalainen E. M. Lappalainen P. (2006). Formins regulate actin filament flexibility through long range allosteric interactions. J. Biol. Chem. 281, 10727–10736. 10.1074/jbc.M510252200 16490788 PMC2865996

[B12] Chen Q. Pollard T. D. (2011). Actin filament severing by cofilin is more important for assembly than constriction of the cytokinetic contractile ring. J. Cell Biol. 195, 485–498. 10.1083/jcb.201103067 22024167 PMC3206353

[B13] Chesarone-Cataldo M. Guérin C. Yu J. H. Wedlich-Soldner R. Blanchoin L. Goode B. L. (2011). The myosin passenger protein Smy1 controls actin cable structure and dynamics by acting as a formin damper. Dev. Cell 21, 217–230. 10.1016/j.devcel.2011.07.004 21839918 PMC3157649

[B14] Chugh P. Clark A. G. Smith M. B. Cassani D. A. D. Dierkes K. Ragab A. (2017). Actin cortex architecture regulates cell surface tension. Nat. Cell Biol. 19, 689–697. 10.1038/ncb3525 28530659 PMC5536221

[B15] Claessens M. M. A. E. Bathe M. Frey E. Bausch A. R. (2006). Actin-binding proteins sensitively mediate F-actin bundle stiffness. Nat. Mater. 5, 748–753. 10.1038/nmat1718 16921360

[B16] Courson D. S. Rock R. S. (2010). Actin cross-link assembly and disassembly mechanics for α-actinin and fascin. J. Biol. Chem. 285, 26350–26357. 10.1074/jbc.M110.123117 20551315 PMC2924060

[B17] Courtemanche N. (2018). Mechanisms of formin-mediated actin assembly and dynamics. Biophys. Rev. 10, 1553–1569. 10.1007/s12551-018-0468-6 30392063 PMC6297096

[B18] De La Cruz E. M. (2005). Cofilin binding to muscle and non-muscle actin filaments: Isoform-dependent cooperative interactions. J. Mol. Biol. 346, 557–564. 10.1016/j.jmb.2004.11.065 15670604

[B19] Ennomani H. Letort G. Guerin C. Martiel J.-L. Cao W. Nedelec F. (2016). Architecture and connectivity govern actin network contractility. Curr. Biol. 26, 616–626. 10.1016/j.cub.2015.12.069 26898468 PMC4959279

[B20] Esue O. Harris E. S. Higgs H. N. Wirtz D. (2008). The filamentous actin cross-linking/bundling activity of mammalian formins. J. Mol. Biol. 384, 324–334. 10.1016/j.jmb.2008.09.043 18835565

[B21] Falzone T. T. Lenz M. Kovar D. R. Gardel M. L. (2012). Assembly kinetics determine the architecture of α-actinin crosslinked F-actin networks. Nat. Commun. 3, 861. 10.1038/ncomms1862 22643888 PMC3563296

[B22] Falzone T. T. Oakes P. W. Sees J. Kovar D. R. Gardel M. L. (2013). Actin assembly factors regulate the gelation kinetics and architecture of F-actin networks. Biophys. J. 104, 1709–1719. 10.1016/j.bpj.2013.01.017 23601318 PMC3628567

[B23] Gressin L. Guillotin A. Guerin C. Blanchoin L. Michelot A. (2015). Architecture dependence of actin filament network disassembly. Curr. Biol. 25, 1437–1447. 10.1016/j.cub.2015.04.011 25913406

[B24] Hampton C. M. Taylor D. W. Taylor K. A. (2007). Novel structures for α-actinin:F-actin interactions and their implications for actin–membrane attachment and tension sensing in the cytoskeleton. J. Mol. Biol. 368, 92–104. 10.1016/j.jmb.2007.01.071 17331538 PMC1919418

[B25] Harris E. S. Rouiller I. Hanein D. Higgs H. N. (2006). Mechanistic differences in actin bundling activity of two mammalian formins, FRL1 and mDia2. J. Biol. Chem. 281, 14383–14392. 10.1074/jbc.m510923200 16556604

[B26] Hashimoto Y. Skacel M. Adams J. C. (2005). Roles of fascin in human carcinoma motility and signaling: Prospects for a novel biomarker? Int. J. Biochem. Cell Biol. 37, 1787–1804. 10.1016/j.biocel.2005.05.004 16002322

[B27] Isambert H. Venier P. Maggs A. C. Fattoum A. Kassab R. Pantaloni D. (1995). Flexibility of actin filaments derived from thermal fluctuations: Effect of bound nucleotide, phalloidin, and muscle regulatory proteins. J. Biol. Chem. 270, 11437–11444. 10.1074/jbc.270.19.11437 7744781

[B28] Jacquemet G. Hamidi H. Ivaska J. (2015). Filopodia in cell adhesion, 3D migration and cancer cell invasion. Curr. Opin. Cell Biol. 36, 23–31. 10.1016/j.ceb.2015.06.007 26186729

[B29] Jaiswal R. Breitsprecher D. Collins A. Corrêa I. R. Xu M.-Q. Goode B. L. (2013). The formin Daam1 and fascin directly collaborate to promote filopodia formation. Curr. Biol. 23, 1373–1379. 10.1016/j.cub.2013.06.013 23850281 PMC3748375

[B30] Jansen S. Collins A. Yang C. Rebowski G. Svitkina T. Dominguez R. (2011). Mechanism of actin filament bundling by fascin. J. Biol. Chem. 286, 30087–30096. 10.1074/jbc.M111.251439 21685497 PMC3191048

[B31] Johnson M. East D. A. Mulvihill D. P. (2014). Formins determine the functional properties of actin filaments in yeast. Curr. Biol. 24, 1525–1530. 10.1016/j.cub.2014.05.034 24954052

[B32] Kliewe F. Scharf C. Rogge H. Darm K. Lindenmeyer M. T. Amann K. (2017). Studying the role of fascin-1 in mechanically stressed podocytes. Sci. Rep. 7, 9916. 10.1038/s41598-017-10116-4 28855604 PMC5577297

[B33] Kovar D. R. Kuhn J. R. Tichy A. L. Pollard T. D. (2003). The fission yeast cytokinesis formin Cdc12p is a barbed end actin filament capping protein gated by profilin. J. Cell Biol. 161, 875–887. 10.1083/jcb.200211078 12796476 PMC2172974

[B34] Kovar D. R. Harris E. S. Mahaffy R. Higgs H. N. Pollard T. D. (2006). Control of the assembly of ATP- and ADP-actin by formins and profilin. Cell 124, 423–435. 10.1016/j.cell.2005.11.038 16439214

[B35] Kuhn J. R. Pollard T. D. (2005). Real-time measurements of actin filament polymerization by total internal reflection fluorescence microscopy. Biophys. J. 88, 1387–1402. 10.1529/biophysj.104.047399 15556992 PMC1305141

[B36] Lappalainen P. (2016). Actin-binding proteins: the long road to understanding the dynamic landscape of cellular actin networks. Mol. Biol. Cell 27, 2519–2522. 10.1091/mbc.E15-10-0728 27528696 PMC4985253

[B37] Li A. Dawson J. C. Forero-Vargas M. Spence H. J. Yu X. König I. (2010). The actin-bundling protein fascin stabilizes actin in invadopodia and potentiates protrusive invasion. Curr. Biol. 20, 339–345. 10.1016/j.cub.2009.12.035 20137952 PMC3163294

[B38] Lizárraga F. Poincloux R. Romao M. Montagnac G. Le Dez G. Bonne I. (2009). Diaphanous-related formins are required for invadopodia formation and invasion of breast tumor cells. Cancer Res. 69, 2792–2800. 10.1158/0008-5472.CAN-08-3709 19276357

[B39] Lu J. Meng W. Poy F. Maiti S. Goode B. L. Eck M. J. (2007). Structure of the FH2 domain of Daam1: Implications for formin regulation of actin assembly. J. Mol. Biol. 369, 1258–1269. 10.1016/j.jmb.2007.04.002 17482208 PMC1939941

[B40] Mallavarapu A. Mitchison T. (1999). Regulated actin cytoskeleton assembly at filopodium tips controls their extension and retraction. J. Cell Biol. 146, 1097–1106. 10.1083/jcb.146.5.1097 10477762 PMC2169471

[B41] Mattila P. K. Lappalainen P. (2008). Filopodia: molecular architecture and cellular functions. Nat. Rev. Mol. Cell Biol. 9, 446–454. 10.1038/nrm2406 18464790

[B42] McCullough B. R. Blanchoin L. Martiel J.-L. De La Cruz E. M. (2008). Cofilin increases the bending flexibility of actin filaments: Implications for severing and cell mechanics. J. Mol. Biol. 381, 550–558. 10.1016/j.jmb.2008.05.055 18617188 PMC2753234

[B43] McGhee J. D. von Hippel P. H. (1974). Theoretical aspects of DNA-protein interactions: co-operative and non-co-operative binding of large ligands to a one-dimensional homogeneous lattice. J. Mol. Biol. 86, 469–489. 10.1016/0022-2836(74)90031-x 4416620

[B44] Mellor H. (2010). The role of formins in filopodia formation. Biochim. Biophys. Acta 1803, 191–200. 10.1016/j.bbamcr.2008.12.018 19171166

[B45] Miao Y. Han X. Zheng L. Xie Y. Mu Y. Yates J. R. (2016). Fimbrin phosphorylation by metaphase Cdk1 regulates actin cable dynamics in budding yeast. Nat. Commun. 7, 11265. 10.1038/ncomms11265 27068241 PMC4832064

[B46] Michelot A. Drubin D. G. (2011). Building distinct actin filament networks in a common cytoplasm. Curr. Biol. 21, R560–R569. 10.1016/j.cub.2011.06.019 21783039 PMC3384529

[B47] Mizuno H. Tanaka K. Yamashiro S. Narita A. Watanabe N. (2018). Helical rotation of the diaphanous-related formin mDia1 generates actin filaments resistant to cofilin. Proc. Natl. Acad. Sci. U. S. A. 115, E5000–E5007. 10.1073/pnas.1803415115 29760064 PMC5984536

[B48] Moseley J. B. Sagot I. Manning A. L. Xu Y. Eck M. J. Pellman D. (2004). A conserved mechanism for Bni1- and mDia1-induced actin assembly and dual regulation of bni1 by Bud6 and profilin. Mol. Biol. Cell 15, 896–907. 10.1091/mbc.e03-08-0621 14657240 PMC329402

[B49] Otomo T. Tomchick D. R. Otomo C. Panchal S. C. Machius M. Rosen M. K. (2005). Structural basis of actin filament nucleation and processive capping by a formin homology 2 domain. Nature 433, 488–494. 10.1038/nature03251 15635372

[B50] Papp G. Bugyi B. Ujfalusi Z. Barko S. Hild G. Somogyi B. (2006). Conformational changes in actin filaments induced by formin binding to the barbed end. Biophys. J. 91, 2564–2572. 10.1529/biophysj.106.087775 16829561 PMC1562385

[B51] Pollard T. D. Cooper J. A. (1984). Quantitative analysis of the effect of Acanthamoeba profilin on actin filament nucleation and elongation. Biochemistry 23, 6631–6641. 10.1021/bi00321a054 6543322

[B52] Pollard T. D. (2016). Actin and actin-binding proteins. Cold Spring Harb. Perspect. Biol. 8, a018226. 10.1101/cshperspect.a018226 26988969 PMC4968159

[B53] Popowicz G. M. Schleicher M. Noegel A. A. Holak T. A. (2006). Filamins: promiscuous organizers of the cytoskeleton. Trends biochem. Sci. 31, 411–419. 10.1016/j.tibs.2006.05.006 16781869

[B54] Pring M. Evangelista M. Boone C. Yang C. Zigmond S. H. (2003). Mechanism of formin-induced nucleation of actin filaments. Biochemistry 42, 486–496. 10.1021/bi026520j 12525176

[B55] Pruyne D. (2016). Revisiting the phylogeny of the animal formins: Two new subtypes, relationships with multiple wing hairs proteins, and a lost human formin. PLoS One 11, e0164067. 10.1371/journal.pone.0164067 27695129 PMC5047451

[B56] Reymann A.-C. Boujemaa-Paterski R. Martiel J.-L. Guérin C. Cao W. Chin H. F. (2012). Actin network architecture can determine myosin motor activity. Science 336, 1310–1314. 10.1126/science.1221708 22679097 PMC3649007

[B57] Schirenbeck A. Bretschneider T. Arasada R. Schleicher M. Faix J. (2005). The Diaphanous-related formin dDia2 is required for the formation and maintenance of filopodia. Nat. Cell Biol. 7, 619–625. 10.1038/ncb1266 15908944

[B58] Schönichen A. Geyer M. (2010). Fifteen formins for an actin filament: a molecular view on the regulation of human formins. Biochim. Biophys. Acta 1803, 152–163. 10.1016/j.bbamcr.2010.01.014 20102729

[B59] Schönichen A. Mannherz H. G. Behrmann E. Mazur A. J. Kühn S. Silván U. (2013). FHOD1 is a combined actin filament capping and bundling factor that selectively associates with actin arcs and stress fibers. J. Cell Sci. 126, 1891–1901. 10.1242/jcs.126706 23444374

[B60] Scott B. J. Neidt E. M. Kovar D. R. (2011). The functionally distinct fission yeast formins have specific actin-assembly properties. Mol. Biol. Cell 22, 3826–3839. 10.1091/mbc.e11-06-0492 21865598 PMC3192862

[B61] Sedeh R. S. Fedorov A. A. Fedorov E. V. Ono S. Matsumura F. Almo S. C. (2010). Structure, evolutionary conservation, and conformational dynamics of *Homo sapiens* fascin-1, an F-actin crosslinking protein. J. Mol. Biol. 400, 589–604. 10.1016/j.jmb.2010.04.043 20434460 PMC7141155

[B62] Sept D. Xu J. Pollard T. D. McCammon J. A. (1999). Annealing accounts for the length of actin filaments formed by spontaneous polymerization. Biophys. J. 77, 2911–2919. 10.1016/s0006-3495(99)77124-9 10585915 PMC1300564

[B63] Sherer L. A. Zweifel M. E. Courtemanche N. (2018). Dissection of two parallel pathways for formin-mediated actin filament elongation. J. Biol. Chem. 293, 17917–17928. 10.1074/jbc.RA118.004845 30266808 PMC6240877

[B64] Sjöblom B. Salmazo A. Djinović-Carugo K. (2008). Alpha-actinin structure and regulation. Cell. Mol. Life Sci. 65, 2688–2701. 10.1007/s00018-008-8080-8 18488141 PMC11131806

[B65] Skau C. T. Neidt E. M. Kovar D. R. (2009). Role of tropomyosin in formin-mediated contractile ring assembly in fission yeast. Mol. Biol. Cell 20, 2160–2173. 10.1091/mbc.e08-12-1201 19244341 PMC2669024

[B66] Spudich J. A. Watt S. (1971). The regulation of rabbit skeletal muscle contraction: I. biochemical studies of the interaction of the tropomyosin-troponin complex with actin and the proteolytic fragments of myosin. J. Biol. Chem. 246, 4866–4871. 10.1016/S0021-9258(18)62016-2 4254541

[B67] Stam S. Freedman S. L. Banerjee S. Weirich K. L. Dinner A. R. Gardel M. L. (2017). Filament rigidity and connectivity tune the deformation modes of active biopolymer networks. Proc. Natl. Acad. Sci. U. S. A. 114, E10037–E10045. 10.1073/pnas.1708625114 29114058 PMC5703288

[B68] Suzuki E. L. Chikireddy J. Dmitrieff S. Guichard B. Romet-Lemonne G. Jégou A. (2020). Geometrical constraints greatly hinder formin mDia1 activity. Nano Lett. 20, 22–32. 10.1021/acs.nanolett.9b02241 31797667 PMC7086397

[B69] Svitkina T. (2018). The actin cytoskeleton and actin-based motility. Cold Spring Harb. Perspect. Biol. 10, a018267. 10.1101/cshperspect.a018267 29295889 PMC5749151

[B70] Tang H. Bidone T. C. Vavylonis D. (2015). Computational model of polarized actin cables and cytokinetic actin ring formation in budding yeast. Cytoskelet. Hob. 72, 517–533. 10.1002/cm.21258 PMC4715483 26538307

[B71] Van Audenhove I. Denert M. Boucherie C. Pieters L. Cornelissen M. Gettemans J. (2016). Fascin rigidity and L-plastin flexibility cooperate in cancer cell invadopodia and filopodia. J. Biol. Chem. 291, 9148–9160. 10.1074/jbc.M115.706937 26945069 PMC4861481

[B72] Vavylonis D. Kovar D. R. O’Shaughnessy B. Pollard T. D. (2006). Model of formin-associated actin filament elongation. Mol. Cell 21, 455–466. 10.1016/j.molcel.2006.01.016 16483928 PMC3716371

[B73] Vavylonis D. Wu J.-Q. Hao S. O’Shaughnessy B. Pollard T. D. (2008). Assembly mechanism of the contractile ring for cytokinesis by fission yeast. Science 319, 97–100. 10.1126/science.1151086 18079366

[B74] Vignjevic D. Kojima S. Aratyn Y. Danciu O. Svitkina T. Borisy G. G. (2006). Role of fascin in filopodial protrusion. J. Cell Biol. 174, 863–875. 10.1083/jcb.200603013 16966425 PMC2064340

[B75] Weaver A. M. (2006). Invadopodia: specialized cell structures for cancer invasion. Clin. Exp. Metastasis 23, 97–105. 10.1007/s10585-006-9014-1 16830222

[B76] Winkelman J. D. Bilancia C. G. Peifer M. Kovar D. R. (2014). Ena/VASP Enabled is a highly processive actin polymerase tailored to self-assemble parallel-bundled F-actin networks with Fascin. Proc. Natl. Acad. Sci. U. S. A. 111, 4121–4126. 10.1073/pnas.1322093111 24591594 PMC3964058

[B77] Winkelman J. D. Suarez C. Hocky G. M. Harker A. J. Morganthaler A. N. Christensen J. R. (2016). Fascin- and α-actinin-bundled networks contain intrinsic structural features that drive protein sorting. Curr. Biol. 26, 2697–2706. 10.1016/j.cub.2016.07.080 27666967 PMC5119644

[B78] Yamakita Y. Ono S. Matsumura F. Yamashiro S. (1996). Phosphorylation of human fascin inhibits its actin binding and bundling activities. J. Biol. Chem. 271, 12632–12638. 10.1074/jbc.271.21.12632 8647875

[B79] Yamakita Y. Matsumura F. Lipscomb M. W. Chou P. Werlen G. Burkhardt J. K. (2011). Fascin1 promotes cell migration of mature dendritic cells. J. Immunol. 186, 2850–2859. 10.4049/jimmunol.1001667 21263068 PMC3526951

[B80] Yamashiro-Matsumura S. Matsumura F. (1985). Purification and characterization of an F-actin-bundling 55-kilodalton protein from HeLa cells. J. Biol. Chem. 260, 5087–5097. 10.1016/S0021-9258(18)89183-9 3886649

[B81] Yamashita M. Higashi T. Suetsugu S. Sato Y. Ikeda T. Shirakawa R. (2007). Crystal structure of human DAAM1 formin homology 2 domain. Genes Cells. 12, 1255–1265. 10.1111/j.1365-2443.2007.01132.x 17986009

[B82] Yang S. Huang F.-K. Huang J. Chen S. Jakoncic J. Leo-Macias A. (2013). Molecular mechanism of fascin function in filopodial formation. J. Biol. Chem. 288, 274–284. 10.1074/jbc.M112.427971 23184945 PMC3537022

[B83] Zweifel M. E. Courtemanche N. (2020). Competition for delivery of profilin–actin to barbed ends limits the rate of formin-mediated actin filament elongation. J. Biol. Chem. 295, 4513–4525. 10.1074/jbc.RA119.012000 32075907 PMC7135983

[B84] Zweifel M. E. Sherer L. A. Mahanta B. Courtemanche N. (2021). Nucleation limits the lengths of actin filaments assembled by formin. Biophys. J. 120, 4442–4456. 10.1016/j.bpj.2021.09.003 34506773 PMC8553668

