# Supplementary material for: Cooperative bundling by fascin generates actin structures with architectures that depend on filament length
Source: Front Cell Dev Biol. 2022 Sep 2;10:974047. doi: 10.3389/fcell.2022.974047 (PMC9479110; doi:10.3389/fcell.2022.974047)
Supplement: Supplementary file 3 [file DataSheet1.docx]

Supplementary Material

# Supplementary Figures

**Supplementary Figure S1. Cdc12p increases the number of actin filaments assembled during polymerization reactions**. 3 µM actin monomers were polymerized in the absence (left) or presence (right) of 750 nM Cdc12p, labeled with FITC-phalloidin, and visualized by TIRF microscopy.

**Supplementary Figure S2. Fascin efficiently bundles actin filaments with free and Cdc12p-bound barbed ends.** Co-sedimentation assays measuring fascin-mediated bundling of actin filaments. Filaments were assembled from 2 µM actin monomers in the absence (Actin Alone) or presence of 250 nM Cdc12p (Cdc12p-Assembled) and incubated with 1000 nM fascin for 30 min at 22 ºC. Reactions were spun at low speed (10,000 x *g* or 20,000 x *g* for filaments assembled in the absence and presence of Cdc12p, respectively) to pellet bundles. (A) Representative SDS-PAGE gels showing the supernatant (S) and pellet (P) from reactions containing spontaneously- (left) and Cdc12p-assembled (right) actin filaments. (B) Quantification of the fraction of the total actin contained in each reaction that sediments into the pellet and is therefore incorporated into bundles. Error bars are the standard deviation of the mean bundled fraction obtained from at least three independent experiments.

**Supplementary Figure S3. Fascin-mediated bundling is insensitive to phalloidin labeling.** Co-sedimentation assays measuring the binding of a range of concentrations of fascin to FITC-phalloidin-labeled actin filaments (total [actin] = 2 µM in each reaction). After reaching equilibrium, reactions were spun at low speed (10,000 x *g*) to pellet bundles and analyzed by SDS-PAGE. The intensity of the fascin band in the pelleted fraction was divided by the intensity of the actin band in the pelleted (i.e., bundled) fraction for each reaction. The line is a fit of the McGhee-von Hippel cooperative binding model to the data, which yields a binding affinity (K_d_) and cooperativity constant (ω). Error bars are the standard error of the mean values obtained from at least three independent experiments.

**
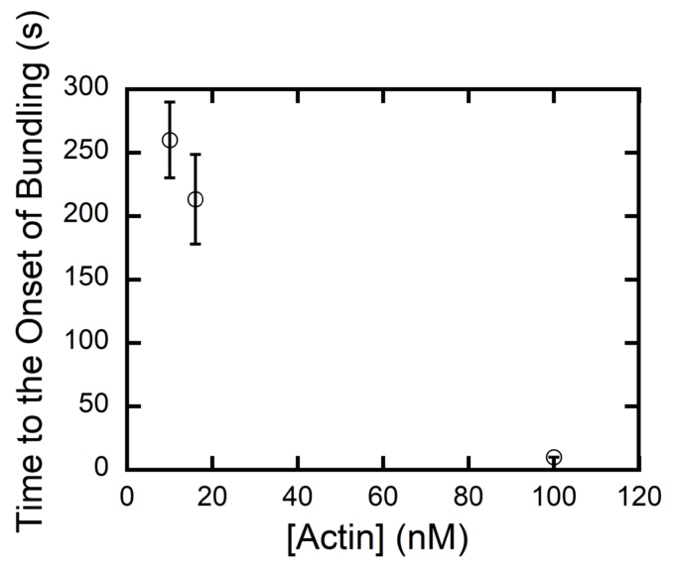
**

**Supplementary Figure S4.** **The length of the delay prior to the onset of bundling depends on the concentration of actin.** Filaments were assembled in the absence of Cdc12p, labeled with FITC-phalloidin, diluted to a range of concentrations, and visualized by TIRF microscopy following the addition of 1000 nM fascin. The length of the delay prior to the onset of bundling was plotted as a function of the concentration of actin. Error bars are the standard error of the mean of three fields of view.

**Supplementary Figure S5.** **Inter-bundle connections form at rates that depend on the filament elongation rate.** Filaments were assembled by Cdc12p (short filaments) or through spontaneous actin polymerization (long filaments), labeled with FITC-phalloidin and imaged in microscopy buffer by TIRF microscopy following the introduction of 1 µM fascin. Preassembled bundles were generated by incubating short filaments with 1 µM fascin prior to the introduction of 0.75 or 1.5 µM actin monomers, 10 µM *S. pombe* profilin, and 1 µM additional fascin. Rates of connection formation were measured by counting the number of instances in which bundles incorporate additional filaments or coalesce with other bundles over the time course of the reaction. Error bars are the standard error of the mean rates of connection formation measured from at least three independent experiments. Asterisks indicate statistical significance (* indicates p < 0.05; “n.s.” indicates p > 0.05). **(A)** Rates at which inter-bundle connections are formed in reactions containing filaments of constant length or elongating bundles. These rates include both short-lived connections that break as elongation and crosslinking progress and stable connections that persist without breaking throughout the course of the reaction. **(B)** Rates at which unstable connections (i.e., connections that break as elongation and crosslinking progress) are formed in reactions containing elongating bundles.

**2 Supplementary Movies**

**Supplementary Movie S1. Fascin-mediated bundling of long filaments.** Filaments were assembled from 2 µM actin monomers, labeled with FITC-phalloidin and visualized by TIRF microscopy at 10-second intervals following the addition of 1400 nM fascin. *Left,* Sequential micrographs spanning 800 seconds of a representative bundling reaction are shown at a rate of 5 frames per second. The dimensions of the micrographs are 80 x 80 µm. *Right*, Automated detection of stretches of bundled (yellow) and single (magenta) filaments over the course of the reaction.

**Supplementary Movie S2. Fascin-mediated bundling of short filaments.** Filaments were assembled from 2 µM actin monomers in the presence of 85 nM Cdc12p, labeled with FITC-phalloidin and visualized by TIRF microscopy at 10-second intervals following the addition of 1000 nM fascin. *Left,* Sequential micrographs spanning 800 seconds of a representative bundling reaction are shown at a rate of 5 frames per second. The dimensions of the micrographs are 80 x 80 µm. *Right*, Automated detection of stretches of bundled (yellow) and single (magenta) filaments over the course of the reaction.
